# Mouse-Adapted SARS-CoV-2 MA10 Strain Displays Differential Pulmonary Tropism and Accelerated Viral Replication, Neurodissemination, and Pulmonary Host Responses in K18-hACE2 Mice

**DOI:** 10.1128/msphere.00558-22

**Published:** 2023-02-02

**Authors:** Côme J. Thieulent, Wellesley Dittmar, Udeni B. R. Balasuriya, Nicholas A. Crossland, Xue Wen, Juergen A. Richt, Mariano Carossino

**Affiliations:** a Department of Pathobiological Sciences, School of Veterinary Medicine, Louisiana State University, Baton Rouge, Louisiana, USA; b Louisiana Animal Disease Diagnostic Laboratory, School of Veterinary Medicine, Louisiana State University, Baton Rouge, Louisiana, USA; c National Emerging Infectious Diseases Laboratories (NEIDL), Boston University, Boston, Massachusetts, USA; d Department of Pathology and Laboratory Medicine, Boston University Chobanian and Avedisian School of Medicine, Boston, Massachusetts, USA; e Department of Diagnostic Medicine and Pathobiology, College of Veterinary Medicine, Kansas State University, Manhattan, Kansas, USA; University of Michigan—Ann Arbor

**Keywords:** ACE2-expressing mice, COVID-19, K18-hACE2 mice, MA10, mouse-adapted virus, respiratory infection, SARS-CoV-2, WA1, mACE2, mouse model, viral replication

## Abstract

Several models were developed to study the pathogenicity of severe acute respiratory syndrome coronavirus 2 (SARS-CoV-2) as well as the *in vivo* efficacy of vaccines and therapeutics. Since wild-type mice are naturally resistant to infection by ancestral SARS-CoV-2 strains, several transgenic mouse models expressing human angiotensin-converting enzyme 2 (hACE2) were developed. An alternative approach has been to develop mouse-adapted SARS-CoV-2 strains. Here, we compared the clinical progression, viral replication kinetics and dissemination, pulmonary tropism, and host innate immune response dynamics between the mouse-adapted MA10 strain and its parental strain (USA-WA1/2020) following intranasal inoculation of K18-hACE2 mice, a widely used model. Compared to its parental counterpart, the MA10 strain induced earlier clinical decline with significantly higher viral replication and earlier neurodissemination. Importantly, the MA10 strain also showed a wider tropism, with infection of bronchiolar epithelia. While both SARS-CoV-2 strains induced comparable pulmonary cytokine/chemokine responses, many proinflammatory and monocyte-recruitment chemokines, such as interleukin-6 (IL-6), tumor necrosis factor alpha (TNF-α), IP-10/CXCL10, and MCP-1/CCL2, showed an earlier peak in MA10-infected mice. Furthermore, both strains induced a similar downregulation of murine *Ace2*, with only a transient downregulation of *Tmprss2* and no alterations in *hACE2* expression. Overall, these data demonstrate that in K18-hACE2 mice, the MA10 strain has a pulmonary tropism that more closely resembles SARS-CoV-2 tropism in humans (airways and pneumocytes) than its parental strain. Its rapid replication and neurodissemination and early host pulmonary responses can have a significant impact on the clinical outcomes of infection and are, therefore, critical features to consider for study designs using these strains and mouse model.

**IMPORTANCE** The COVID-19 pandemic, caused by SARS-CoV-2, is still significantly impacting health care systems around the globe. Refined animal models are needed to study SARS-CoV-2 pathogenicity as well as efficacy of vaccines and therapeutics. In line with this, thorough evaluation of animal models and virus strains/variants are paramount for standardization and meaningful comparisons. Here, we demonstrated differences in replication dynamics between the Wuhan-like USA-WA1/2020 strain and the derivative mouse-adapted MA10 strain in K18-hACE2 mice. The MA10 strain showed accelerated viral replication and neurodissemination, differential pulmonary tropism, and earlier pulmonary innate immune responses. The observed differences allow us to better refine experimental designs when considering the use of the MA10 strain in the widely utilized K18-hACE2 murine model.

## INTRODUCTION

Severe acute respiratory syndrome coronavirus 2 (SARS-CoV-2) is an enveloped, positive-sense, single-stranded RNA virus belonging to the *Betacoronavirus* genus (order *Nidovirales*, family *Coronaviridae*) (1, 2). Since the initial case reported at a seafood market in Wuhan, Hubei Province, China in December 2019 (3–5), SARS-CoV-2 has rapidly become a pandemic (6) with 660 million confirmed cases and nearly 6.7 million deaths (1.01%) as of 31 December 2022 (7). To infect the host, the viral spike glycoprotein (S) of SARS-CoV-2 utilizes the human angiotensin-converting enzyme 2 (hACE2) as the major entry receptor (8). After virus binding, priming and cleavage of the S glycoprotein by the host transmembrane serine protease 2 (TMPRSS2), conformational changes in S trimers allow for subsequent fusion of the cellular membrane and viral envelope, facilitating virus entry (8, 9). CD147, neuropilin 1, and the neutral amino acid transporter B0AT1 were also identified as host receptors/factors that may have a potential role in SARS-CoV-2 infection (10–14).

SARS-CoV-2 is the causative agent of coronavirus disease 2019 (COVID-19), causing respiratory disease of variable severity that may lead to the development of acute respiratory distress syndrome (ARDS) (15–17). Although the exact mechanisms by which SARS-CoV-2 induces ARDS are not fully understood, the induction of a cytokine storm, characterized by increased levels of inflammatory cytokines and chemokines, such as interleukin-2 (IL-2), IL-6, IL-10, tumor necrosis factor alpha (TNF-α), granulocyte-macrophage colony-stimulating factor (GM-CSF), monocyte chemoattractant protein-1 (MCP-1/CCL-2), macrophage inflammatory protein 1 alpha (MIP-1α/CCL3), and CXC-chemokine ligand 10 (CXCL10), is considered to be one of the driving factors (18–22).

The low binding affinity between the viral S protein of the ancestral Wuhan-like (USA-WA1/2020) SARS-CoV-2 strains and murine ACE2 (mACE2) renders conventional mouse strains as naturally resistant to infection, which has posed a significant challenge for the development of murine models of COVID-19 (23, 24). This challenge was in part countered with the emergence of variants of concern (VOCs) possessing the N501Y mutation on the S glycoprotein, which increases the S-mACE2 interaction and also allows for infection of airway epithelia (25). Several strategies have also been developed to increase the susceptibility of laboratory mice to SARS-CoV-2 infection such as using transgenic mice expressing hACE2 under the K18 promoter (K18-hACE2) (26–29), transduction of hACE2 using an adenoviral delivery system (30), or using mouse-adapted SARS-CoV-2 strains (31–34). The K18-hACE2 mouse model expresses the hACE2 transgene along with the mACE2 receptor (27) and is a common *in vivo* model used to study SARS-CoV-2 pathogenesis and efficacy of therapeutics and vaccines. The main limitation of this model is its high lethality associated with SARS-CoV-2 neuroinvasion and subsequent clinical decline following intranasal challenge (35–38), limiting long-term studies. The mouse-adapted SARS-CoV-2 MA strain was developed by Dinnon et al. (33) via engineering amino acid residues Q498Y/P499T of the receptor binding domain (RBD) on the S protein involved in the S-mACE2 interface to allow binding of the S glycoprotein to mACE2. To increase the virulence of this engineered virus, Leist et al. (34) developed the mouse-adapted MA10 strain by 10 subsequent passages of the SARS-CoV-2 MA strain in BALB/cAnNHsd mice, resulting in increased virulence in both young and aged BALB/cAnNHsd mice. Importantly, both the MA and MA10 strains demonstrated acquired bronchiolar epithelial tropism, a feature not identified in K18-hACE2 mice infected with the USA-WA1/2020 strain of SARS-CoV-2 (35).

Refined models to study SARS-CoV-2 pathogenicity are still needed, and a potential alternative approach is to use the MA10 strain, which has an expanded cellular tropism, in the transgenic K18-hACE2 mouse model. Although it was recently demonstrated that this mouse-adapted strain induces high lethality in K18-hACE2 mice (39), an extensive pathogenicity study to comparatively identify differences with its parental strain and better inform its use in this humanized mouse model has not yet been conducted. In this study, a time course investigation following intranasal challenge of K18-hACE2 mice with the mouse-adapted MA10 strain was conducted and compared to its parental strain USA-WA1/2020. Clinical outcomes, viral replication kinetics, dissemination, tropism, and histomorphological changes in the lung and brain, pulmonary cytokine/chemokine responses, and pulmonary *mAce2*, *hACE2*, and *Tmprss2* expression dynamics were quantitated and compared. This study highlights the importance of thoroughly characterizing SARS-CoV-2 animal models for preclinical trials to better define relevant experimental observation time points/data collection points and assist in drawing adequate interpretations.

## RESULTS

### SARS-CoV-2 MA10 strain induces earlier and more severe weight loss with comparable lethality to the USA-WA1/2020 parental strain in intranasally infected K18-hACE2 mice.

**FIG 1:**
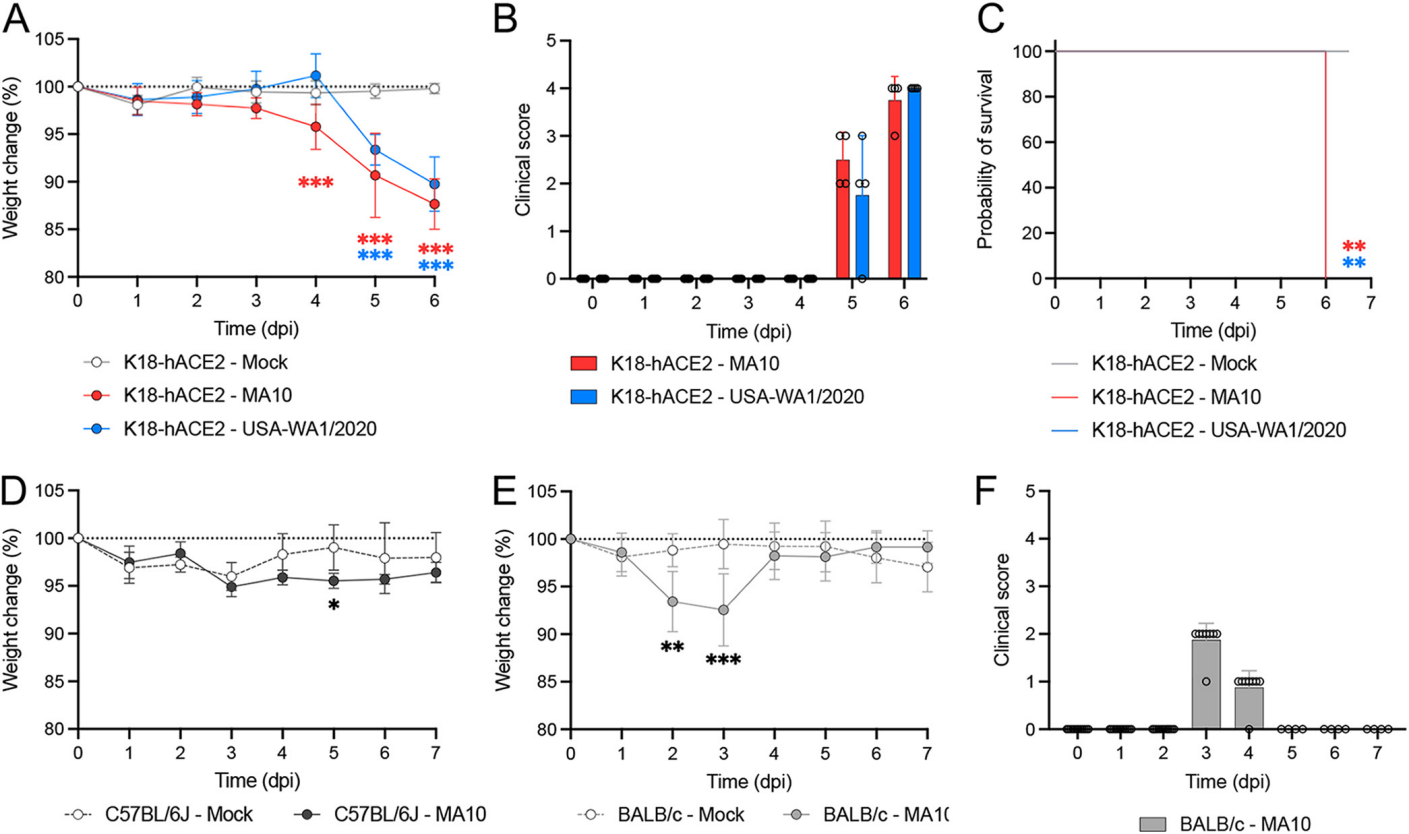
SARS-CoV-2 MA10 and USA-WA1/2020 strains induced weight loss and lethality in K18-hACE2 mice. Mice were intranasally infected with 2 × 10^4^ PFU of SARS-CoV-2 MA10 (red; *n* = 12), USA-WA1/2020 strain (blue; *n* = 12), or mock infected with 50 μL of MEM (gray; *n* = 4). Body weight (A), clinical signs (B), and survival (C) of K18-hACE2 mice were monitored daily. Body weight of C57BL/6J (D) and BALB/c (E) mice intranasally infected with 2 × 10^4^ PFU of SARS-CoV-2 MA10. Clinical disease developed in infected BALB/c (F) but not in C57BL/6J mice. Infection of BALB/c and C57BL/6J was nonlethal at this dose, and animals recovered following infection. Points and bars represent the mean ± standard deviation. *, *P *≤ 0.05; **, *P *≤ 0.01; ***, *P *≤* *0.001.

K18-hACE2 mice were intranasally infected with 2 × 10^4^ PFU of either the SARS-CoV-2 MA10 strain or its parental strain USA-WA1/2020 and monitored for 6 to 7 days, at which point euthanasia criteria was met in all animals. Significant weight loss was first observed in the MA10-infected mice at 4 days postinfection (dpi) (4.22 ± 2.37%; *P ≤ *0.001), and body weight continued to decrease throughout the remainder of the experiment (Fig. 1A). Significant weight loss in mice infected with the same dose of SARS-CoV-2 USA-WA1/2020 strain was first identified at 5 dpi instead (6.63 ± 1.61%; *P* ≤ 0.001). Peak in weight loss occurred at 6 dpi for both MA10- and USA-WA1/2020-infected mice with 12.34 ± 2.64% (*P *≤* *0.001) and 10.23 ± 2.86% (*P ≤ *0.001) loss, respectively. Besides weight loss, clinical signs (increased respiratory rate/effort, reluctance to move, tremors, and/or poor responsiveness) were initially noted at 5 dpi for both groups, with a maximum score reached at 6 dpi (Fig. 1B), time at which humane euthanasia endpoint was reached (see Fig. 6C). Altogether, these data demonstrate that the MA10 strain of SARS-CoV-2 induces clinical decline at least 24 h earlier than the ancestral USA-WA1/2020 strain in K18-hACE2 mice.

Comparatively, MA10-infected C57BL/6J mice, the background mouse strain of the K18-hACE2 mice, and BALB/c mice showed significant and transient weight loss at 5 dpi (2.58 ± 0.81%; *P = *0.029) or as early as 2 dpi (5.41 ± 3.17%; *P ≤ *0.001), respectively (Fig. 1D and E). Clinical signs (ruffled fur and lethargy) were observed at 3 and 4 dpi (Fig. 1F) in infected BALB/c mice but not in infected C57BL/6J mice (data not shown). All BALB/c infected mice recovered by 5 dpi (Fig. 1E and F), and no lethality was recorded in either infected BALB/c or in C57BL/6J mice by 7 dpi (data not shown).

### SARS-CoV-2 MA10 strain induces rapid replication and an earlier peak in viral titers in the lung of K18-hACE2 mice compared to that of the SARS-CoV-2 USA-WA1/2020 parental strain.

**FIG 2:**
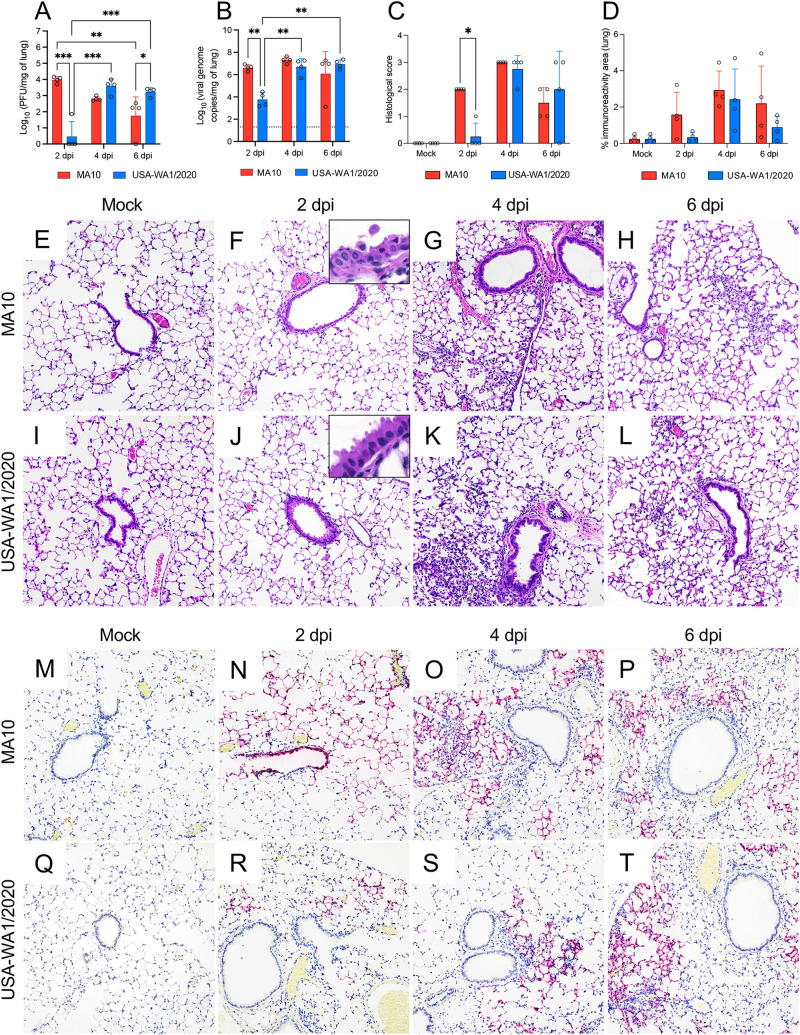
Comparative temporal analysis of SARS-CoV-2 MA10 and USA-WA1/2020 replication and pathological alterations in the lung of K18-hACE2 mice. Infectious viral particles (A) and viral RNA (B) were quantified in the lung of infected mice at 2, 4, and 6 dpi; the MA10-infected mice showed significantly higher viral titers and viral RNA in the lung by 2 dpi compared to the parental strain. Histological scores (C) and percentage of SARS-CoV-2 immunoreactivity measured by quantitative IHC (D) in the lung of K18-hACE2 mice. Histopathological alterations in the lung appear earlier in the MA10-infected mice compared to the parental strain. Bars represent the mean ± standard deviation. Dotted line represents the limit of detection. Temporal histologic lesions (E to L) and viral antigen abundance and distribution (M to T) in the lung of MA10- and USA-WA1/2020-infected K18-hACE2 mice. Bronchiolar epithelial degeneration and necrosis with viral antigen located within bronchiolar epithelium were evident in the MA10-infected group (F and N, and inset) at 2 dpi but not in the USA-WA1/2020-infected group (J and R, and inset). Mild-to-moderate pneumonia with a predominantly mononuclear cell infiltrate were evident at 4 dpi and persisted at 6 dpi in both infected groups (G, H, K, and L), with abundant viral antigen within AT1 and AT2 cells (M to T). H&E (E to L) and Fast Red (viral antigen) (M to T); ×200 total magnification. *, *P *≤* *0.05; **, *P *≤ 0.01; ***, *P* ≤ 0.001.

To evaluate viral replication kinetics, four infected K18-hACE2 mice per group were euthanized at 2, 4, and 6 dpi, and the level of infectious virus and viral RNA in the lung were quantitated. At 2 dpi, infectious virus was detectable in the lungs of all MA10-infected K18-hACE2 mice, whereas only one-quarter of mice infected with USA-WA1/2020 had detectable virus (Fig. 2A). Comparatively, viral titers were significantly higher in the lung of MA10-infected K18-hACE2 mice (*P *≤* *0.001), with a >3 log difference compared to the USA-WA1/2020-infected K18-hACE2 mice (Fig. 2A). Viral titers for USA-WA1/2020-infected K18-hACE2 mice reached comparable values to those in the MA10-infected group (2 dpi) only at 4 dpi and thereafter. At 6 dpi, a significantly higher viral titer was observed in the lung of mice infected with the USA-WA1/2020 parental strain compared with the MA10 strain (*P = *0.047). Viral genomic RNA showed similar dynamics during infection, with a rapid spike at 2 dpi for the MA10-infected K18-hACE2 mice, which displayed an ~2.8 log increase compared to the USA-WA1/2020-infected mice (*P = *0.004) (Fig. 2B). These results illustrate accelerated replication dynamics of the MA10 strain in the lungs of K18-hACE2 infected mice compared to the WA1/2020 parental strain.

**FIG 3:**
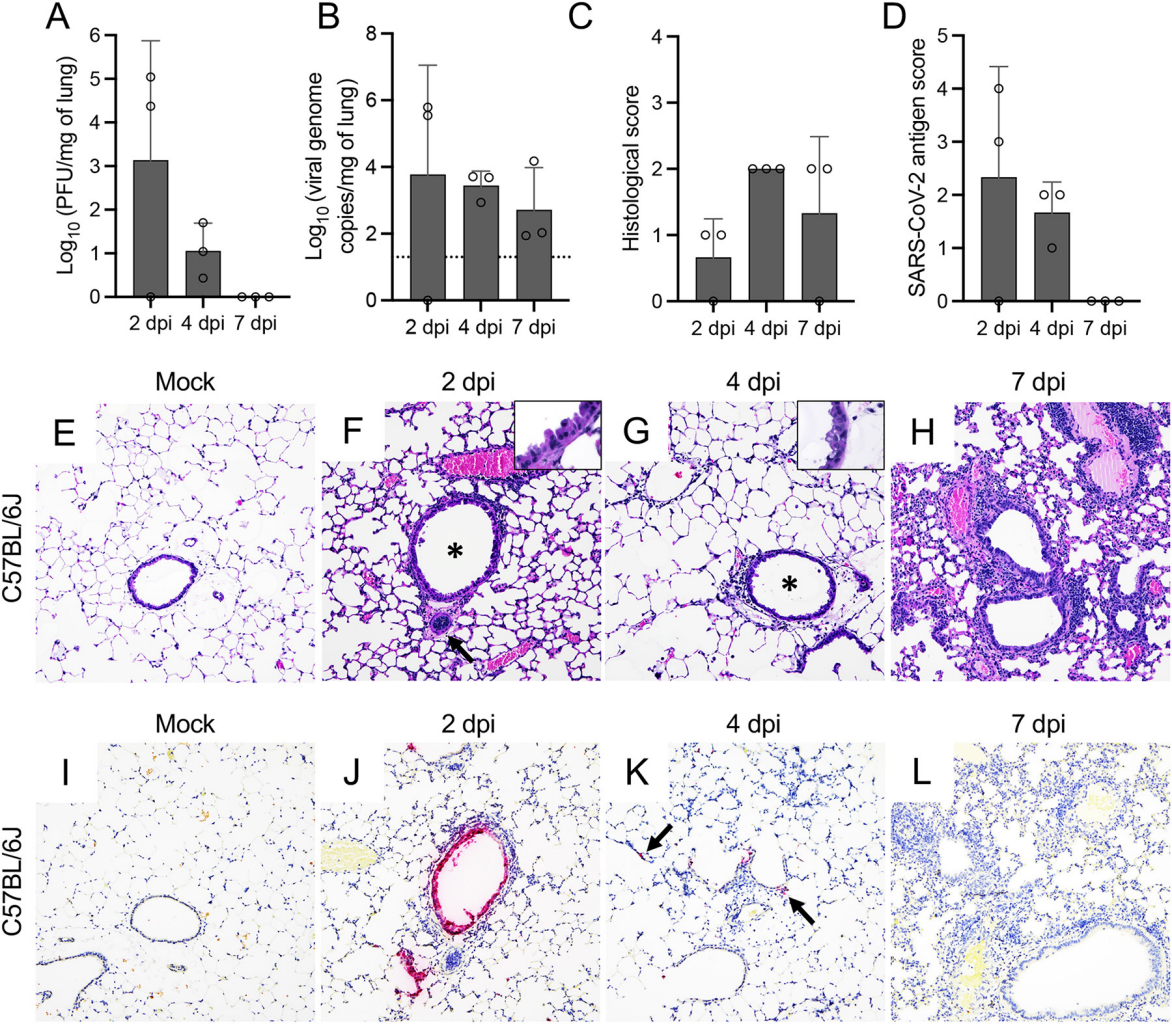
SARS-CoV-2 MA10 infection of C57BL/6J mice. Infectious viral particles (A) and viral RNA (B) were quantified in the lung of infected C57BL/6J mice at 2, 4, and 7 dpi. Histological scores (C) and scoring for SARS-CoV-2 antigen abundance (D) in the lung of C57BL/6J mice are shown. Bars represent the mean ± standard deviation. Dotted line represents the limit of detection. Temporal histologic lesions (E to H) and viral antigen abundance and distribution (I to L) in the lung of MA10-infected C57BL/6J mice. At 2 dpi, there was evidence of bronchiolar (F, asterisk) epithelial degeneration and necrosis (F, inset), with abundant viral antigen (J), mild peribronchiolar inflammation, and blood vessels with hypertrophied (activated) endothelium (F, arrow). Similar alterations were noted at 4 dpi (G, inset), but these resolved by 7 dpi (H). Mild interstitial pneumonia was first identified at 4 dpi (G) and persisted until the end of the study (7 dpi) (H). At 4 dpi, viral antigen was rare and only identified within the cytoplasm of scattered pneumocytes (K, arrows). No viral antigen was noted at 7 dpi (L). H&E (E to H) and Fast Red (viral antigen) (I to L); ×200 total magnification. *, *P *≤* *0.05; **, *P *≤* *0.01; ***, *P *≤ 0.001.

**FIG 4:**
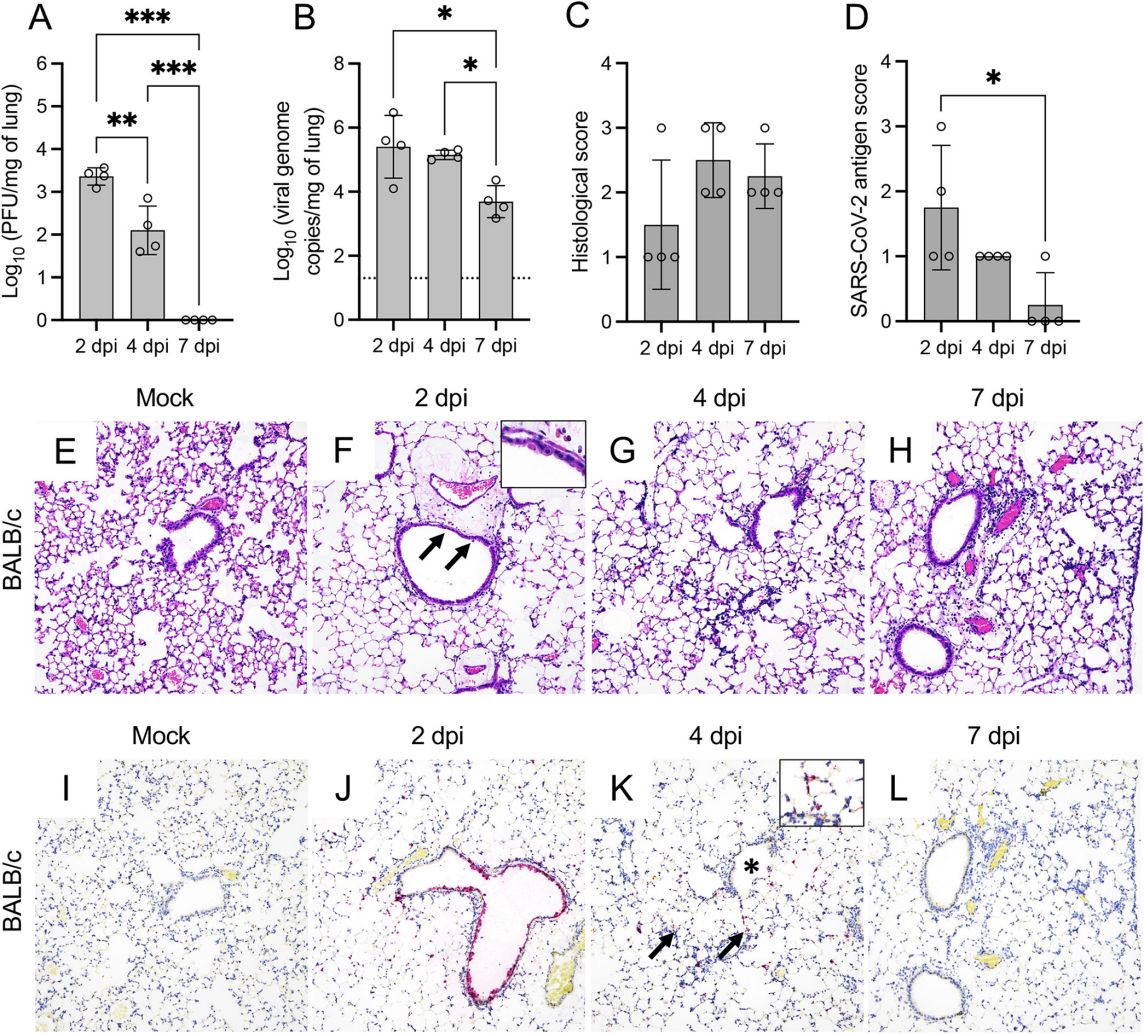
SARS-CoV-2 MA10 induces injury in the lung of BALB/c mice. Infectious viral particles (A) and viral RNA (B) were quantified in the lung of infected BALB/c mice at 2, 4, and 7 dpi. Histological scores (C) and scoring of SARS-CoV-2 antigen abundance (D) in the lung of BALB/c mice are presented. Bars represent the mean ± standard deviation. Dotted line represents the limit of detection. Temporal histologic lesions (E to H) and viral antigen abundance and distribution (I to L) in the lung of MA10-infected BALB/c mice. There is evidence of bronchiolar degeneration/necrosis at 2 dpi (F, arrows and inset), with abundant viral antigen (J) and mild peribronchiolar inflammation. At 4 dpi, there is evidence of interstitial pneumonia with alveolar septa expanded by mononuclear inflammatory cells, and alveolar spaces contain similar inflammatory cells and karyorrhectic debris (G). Similar histologic changes persist at 7 dpi (H). At 4 dpi, viral antigen is mostly frequent in the cytoplasm of AT2 cells within areas of pneumonia (K, arrows and inset) but spare the bronchiolar epithelium (K, asterisk). Viral antigen is rarely detected at 7 dpi (L). H&E (E to H) and Fast Red (viral antigen) (I to L); ×200 total magnification. *, *P *≤* *0.05; **, *P *≤* *0.01; ***, *P *≤* *0.001.

Infectious virus/viral RNA were detectable in the lungs of two out of three of the MA10-infected C57BL/6J mice at 2 dpi and in the lungs of all C57BL/6J mice at 4 dpi (Fig. 3A and B). Even though MA10 viral RNA was detected in the lung at 7 dpi, no infectious virus was recovered. Infectious virus was detectable in the lungs of all BALB/c infected mice at 2 and 4 dpi but not at 7 dpi (Fig. 4A). In contrast, viral RNA was detected in all infected BALB/c mice at all time points (Fig. 4B). Peak in viral titers and viral RNA in these mice occurred at 2 dpi.

### Differential tropism of the SARS-CoV-2 MA10 strain compared to that of the USA-WA1/2020 parental strain is associated with bronchiolitis and subsequent interstitial pneumonia.

To comprehensively characterize differences between these strains in the lung of K18-hACE2 mice, we evaluated the histologic alterations in both infected groups as well as viral tropism, abundance, and spread of viral antigen via immunohistochemistry (IHC) and quantitative image analysis throughout the course of infection (Fig. 2C and Fig. 2E to T). In the MA10-infected K18-hACE2 mice, histologic alterations were evident at 2 dpi and were mainly characterized by multifocal necrotizing bronchiolitis featuring bronchiolar epithelial vacuolation, rounding and detachment, attenuation, and loss of polarity and cilia accompanied by a minimal infiltrate of peribronchiolar lymphocytes and histiocytes (Fig. 2F). In comparison, only sporadic perivascular cuffs composed of minimal lymphocytes were noted in the USA-WA1/2020-infected mice at this time point, with no evidence of airway damage (Fig. 2J). At 4 dpi, all MA10 and USA-WA1/2020-infected K18-hACE2 mice showed evidence of comparable mild to moderate multifocal interstitial pneumonia with predominantly mononuclear cells (lymphocytes and histiocytes) (Fig. 2G and K). In the MA10-infected K18-hACE2 mice, the bronchiolar epithelium was fully regenerated, and no changes were noted in the airways of USA-WA1/2020-infected K18-hACE2 mice as also noted in previous reports (35). Interstitial pneumonia variably persisted until the end of the experiment (6 dpi) (Fig. 2H and L). Histologic lesions were semiquantitatively scored based on their severity (Fig. 2C); the significant difference identified at 2 dpi between MA10- and USA-WA1/2020-infected K18-hACE2 mice is attributable to the bronchiolitis that developed exclusively in the former group at 2 dpi (*P = *0.029) (Fig. 2F).

We subsequently compared viral tropism of these two strains and quantitatively determined the abundance of SARS-CoV-2 N antigen in the lungs via IHC (Fig. 2D and Fig. 2M to T). Correlating with the histologic changes noted at 2 dpi and accelerated elevation of viral titers in the MA10-infected mice, intracytoplasmic viral antigen was abundant within the affected bronchiolar epithelium as well as within neighboring alveolar type 1 and 2 (AT1 and AT2) cells (Fig. 2N). On subsequent time points, viral antigen was limited to the cytoplasm of only AT1 and AT2 cells (Fig. 2O and P). Comparatively and as previously described, USA-WA1/2020 showed exclusive tropism for AT1 and AT2 cells, with no viral antigen detected within bronchiolar epithelium at any time point (Fig. 2R to T). To determine differences in viral spread/abundance between these two strains, we quantitated the immunoreactive area as a percentage of the entire pulmonary parenchyma evaluated (Fig. 2D). Although not statistically significant, a trend similar to viral load data was noted, with a higher immunoreactive area for SARS-CoV-2 N antigen in MA10-infected K18-hACE2 mice at 2 dpi than in USA-WA1/2020-infected animals that subsequently becomes similar at 4 and 6 dpi.

Similar to MA10-infected K18-hACE2 mice, histologic lesions in MA10-infected C57BL/6J mice were initially evident at 2 dpi and were characterized by necrotizing bronchiolitis with abundant epithelial viral antigen and mild peribronchiolar/perivascular mononuclear cell infiltration (Fig. 3C, F, and J). This was subsequently followed by an increase in peribronchiolar and perivascular inflammatory cells at 4 dpi and mild interstitial pneumonia noted both at 4 and 7 dpi (Fig. 3G and H). By 7 dpi, bronchiolar epithelial histomorphological changes were resolved, and viral antigen was no longer detected (Fig. 3L). Histologic changes in the lungs of MA10-infected BALB/c mice were similar (Fig. 4C and Fig. 4E to H), with more frequent foci of interstitial pneumonia and intra-alveolar debris than C57BL/6J mice (Fig. 4G and H). At 2 dpi, viral antigen was detected in the bronchiolar epithelium and was more frequently within alveolar epithelial cells than in MA10-infected C57BL/6J but not as widespread as in infected K18-hACE2 mice (Fig. 4J). At 4 dpi, no viral antigen was detected in the bronchiolar epithelium but persisted within AT2 cells (Fig. 4K to L).

### SARS-CoV-2 MA10 strain exhibits similar neurotropism but accelerated neurodissemination compared to that of the USA-WA1/2020 parental strain in K18-hACE2 mice.

**FIG 5:**
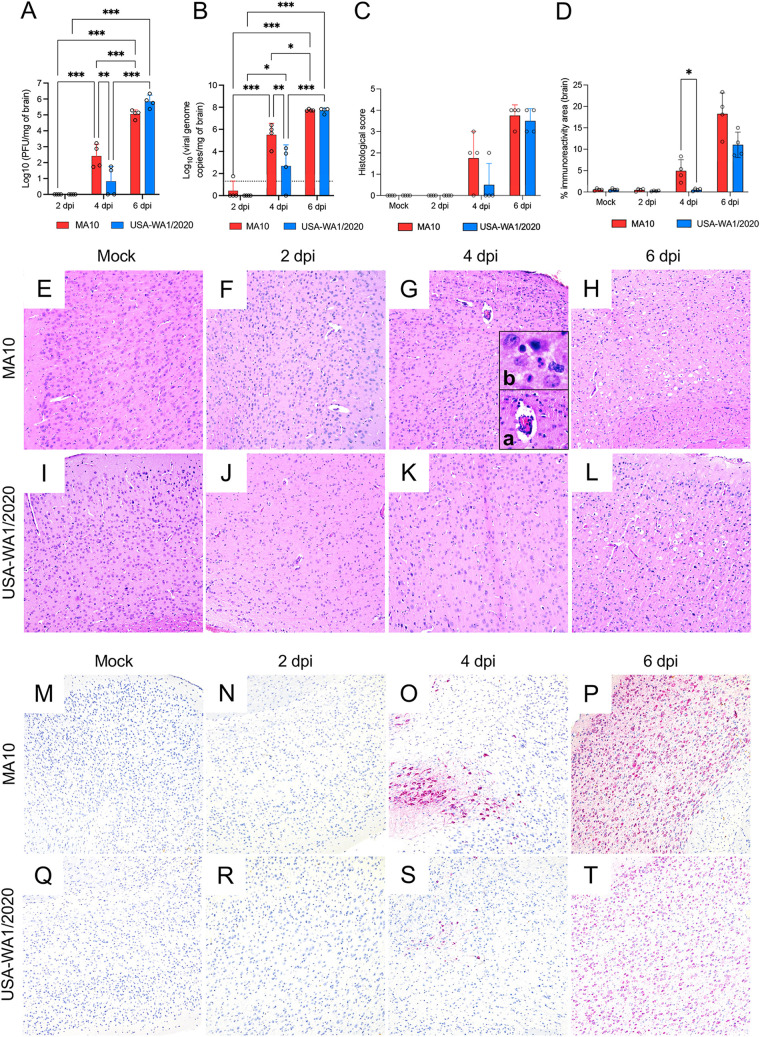
Comparative temporal analysis of SARS-CoV-2 MA10 and USA-WA1/2020 replication and pathological alterations in the brain (cerebral cortex) of K18-hACE2 mice. Infectious viral particles (A) and viral RNA (B) were quantified in the brain of infected mice at 2, 4, and 6 dpi. Histological scores (C) and percentage of SARS-CoV-2 immunoreactivity (D) in the brain of K18-hACE2 mice is shown. Bars represent the mean ± standard deviation. Dotted line represents the limit of detection. Temporal histologic lesions (E to L) and viral antigen abundance and distribution (M to T) in the brain of K18-hACE2 mice. Histologic changes are noted as early as 4 dpi and are more pronounced on the MA10-infected group (G) with delicate perivascular lymphocytic cuffs (G, inset a), sporadic pyknotic nuclei, and increased microglial cells and scattered neutrophils within the neuroparenchyma (G, inset b). At this time point, viral antigen is significantly more abundant in the MA10-infected group compared to that of the group infected with the parental USA-WA1/2020 strain. As previously reported, neuronal vacuolation/necrosis are prominent at 6 dpi (H and L) with diffuse and abundant expression of viral antigen within neuronal bodies and processes (P and T) with cerebellar sparing as previously reported. H&E (E to H) and Fast Red (viral antigen) (I to L); ×200 total magnification. *, *P *≤* *0.05; **, *P *≤ 0.01; ***, *P *≤ 0.001.

The temporal infection dynamics in the brain of both MA10- and USA-WA1/2020-infected K18-hACE2 mice were comparatively evaluated. At 2 dpi, no detectable infectious virus was identified in the brain of either group (Fig. 5A), with a marginal amount of viral RNA detected in the brain of a single MA10-infected mouse (Fig. 5B). At 4 dpi, infectious virus and viral RNA were detected in the brain of all MA10-infected mice but only in 2 of 4 and 3 of 4 K18-hACE2 mice infected with the USA-WA1/2020 strain, respectively. At 4 dpi, viral titers and viral RNA copies per milligram of brain were significantly higher in the MA10-infected K18-hACE2 mice than in the USA-WA1/2020-infected animals (~1.6 log, *P = *0.006; ~2.8 log, *P = *0.007, respectively). Viral titers and viral RNA copies per milligram of brain reached their peak at 6 dpi, with comparable levels between both groups.

In addition, we analyzed morphological changes and viral tropism and spread as described for the lung. No morphological changes or viral antigen were observed in the brain of either group at 2 dpi (Fig. 5C, D, F, J, N, and R). At 4 dpi, histologic alterations were initially seen in both groups of mice, characterized by sporadic neuronal necrosis and microgliosis with occasional lymphocytic perivascular cuffs and few neutrophils within the parenchyma (Fig. 5G and K); these alterations were more pronounced in the MA10-infected animals with identifiable alterations in 3 of 4 animals compared to only 1 of 4 animals in the USA-WA1/2020 group. Viral antigen was detected within clusters of neuronal bodies and neuronal processes within the anterior olfactory nucleus and orbital area of the cerebral cortex as well as the mitral layer of the olfactory bulb in all MA10-infected K18-hACE2 mice and only 1 of 4 USA-WA1/2020-infected mice (Fig. 5O and S). Immunoreactive area quantification revealed a significantly higher SARS-CoV-2 N immunoreactive area in the MA10-infected group than in USA-WA1/2020-infected K18-hACE2 mice (*P = *0.029) (Fig. 5D), indicating more rapid neurodissemination. At 6 dpi, abundant viral antigen was diffusely detected in neurons (with sparing of the cerebellum) in both groups (Fig. 5P and T), accompanied by neuronal vacuolation and necrosis (Fig. 5H and L) as previously described (35). The results show that the MA10 strain retains neurotropism and neuro disseminates occurs more rapidly compared to the USA-WA1/2020 parental strain.

No infectious virus, viral RNA, viral antigen, or histological alterations were detected in the brain of MA10-infected C57BL/6J and BALB/c mice throughout study time points (data not shown), confirming the absence of neurodissemination in these mouse models.

### Pulmonary host cytokine and chemokine responses are temporally delayed in USA-WA1/2020-infected K18-hACE2 mice.

**FIG 6:**
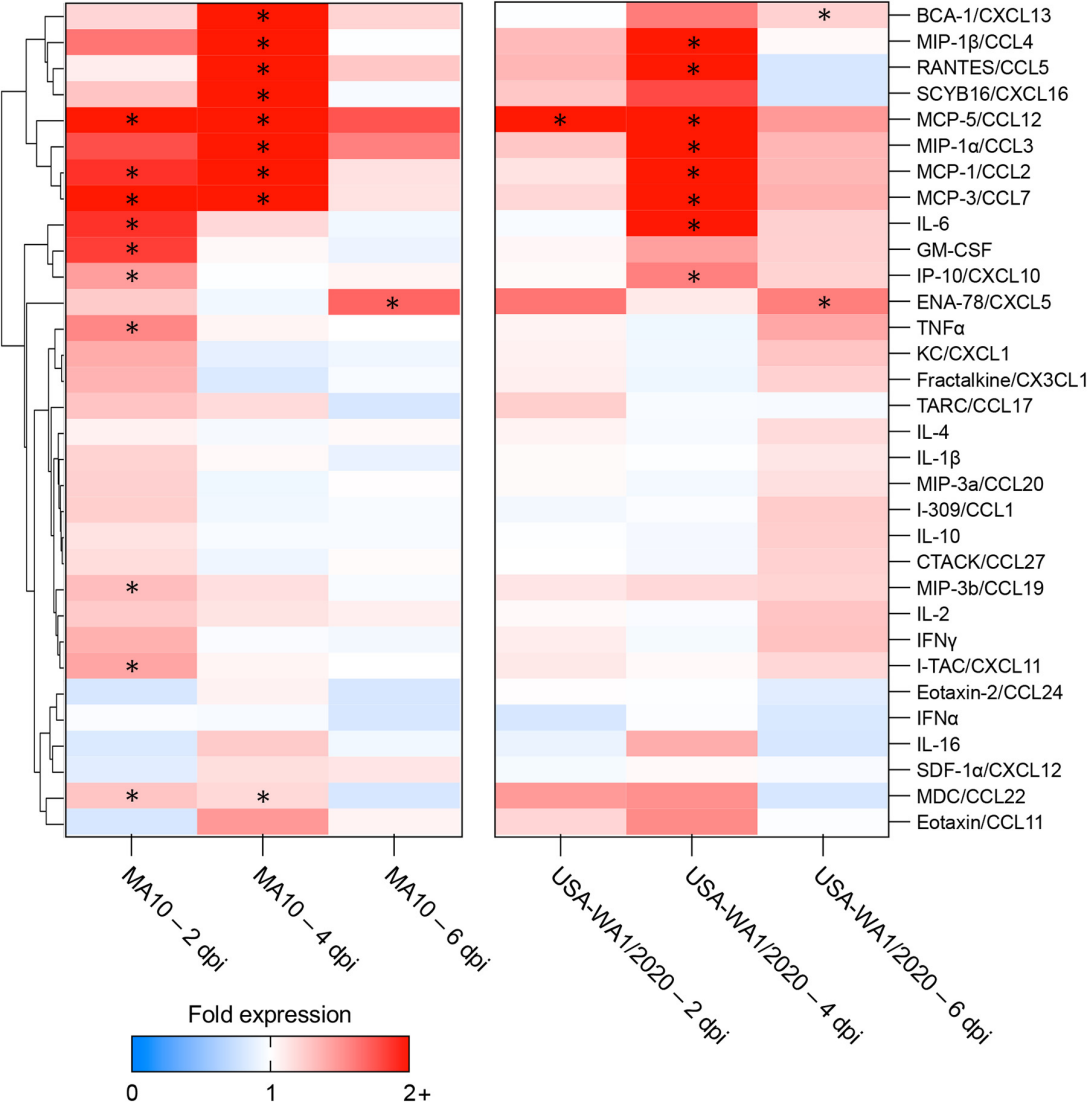
Cytokine and chemokine profiling in the lungs of MA10- and USA-WA1/2020-infected K18-hACE2 mice. The proinflammatory cytokine and chemokine response occurs more rapidly in the lung of K18-hACE2 mice infected with the MA10 strain compared to those infected with USA-WA1/2020 strain. Heatmap depicting the fold change of protein expression in the lung of infected mice at 2, 4, and 6 dpi compared to mock infected K18-hACE2 mice. Color scale depicts differences in fold expression. Cytokines and chemokines that are significantly upregulated compared to the mock are marked with an asterisk.

Severe SARS-CoV-2 infections are associated with a cytokine storm in the lung of patients, mainly characterized by increased expression of proinflammatory cytokines (18–20). The protein expression levels of 32 cytokines were evaluated in the lung of K18-hACE2 mice infected with SARS-CoV-2 MA10 and USA-WA1/2020 strains (see Fig. S1 in the supplemental material). Even though 16 cytokines were significantly upregulated in the lung of MA10-infected K18-hACE2 mice compared to those in mock-infected animals, only 10 were significantly upregulated in the lung of USA-WA1/2020-infected mice (Fig. 6). In addition, the expression of five of the upregulated cytokines/chemokines (BCA-1/CXCL13, MCP-1/CCL2, MCP-3/CCL7, IL-6, and IP-10/CXCL10) was significantly delayed in the lung of USA-WA1/2020-infected K18-hACE2 mice (Fig. 6; see also Fig. S1). No change in alpha interferon (IFN-α) expression was observed in either group, suggestive of inhibition of type I interferons by SARS-CoV-2.

10.1128/msphere.00558-22.1 FIG S1 SARS-CoV-2 MA10 strain induces an early expression of proinflammatory cytokines and chemokines in the lungs of K18-hACE2 mice compared to that of its parental strain USA-WA1/2020. Protein expression of 32 cytokines/chemokines was measured in the lung of mock-, MA10-, and USA-WA1/2020-infected K18-hACE2 mice at 2 dpi, 4 dpi, and 6 dpi (*n* = 4). Data analyzed by nonparametric Wilcoxon tests. Download FIG S1, TIF file, 1.0 MB. Copyright © 2023 Thieulent et al. 2023 Thieulent et al. https://creativecommons.org/licenses/by/4.0/ This content is distributed under the terms of the Creative Commons Attribution 4.0 International license.

**FIG 7:**
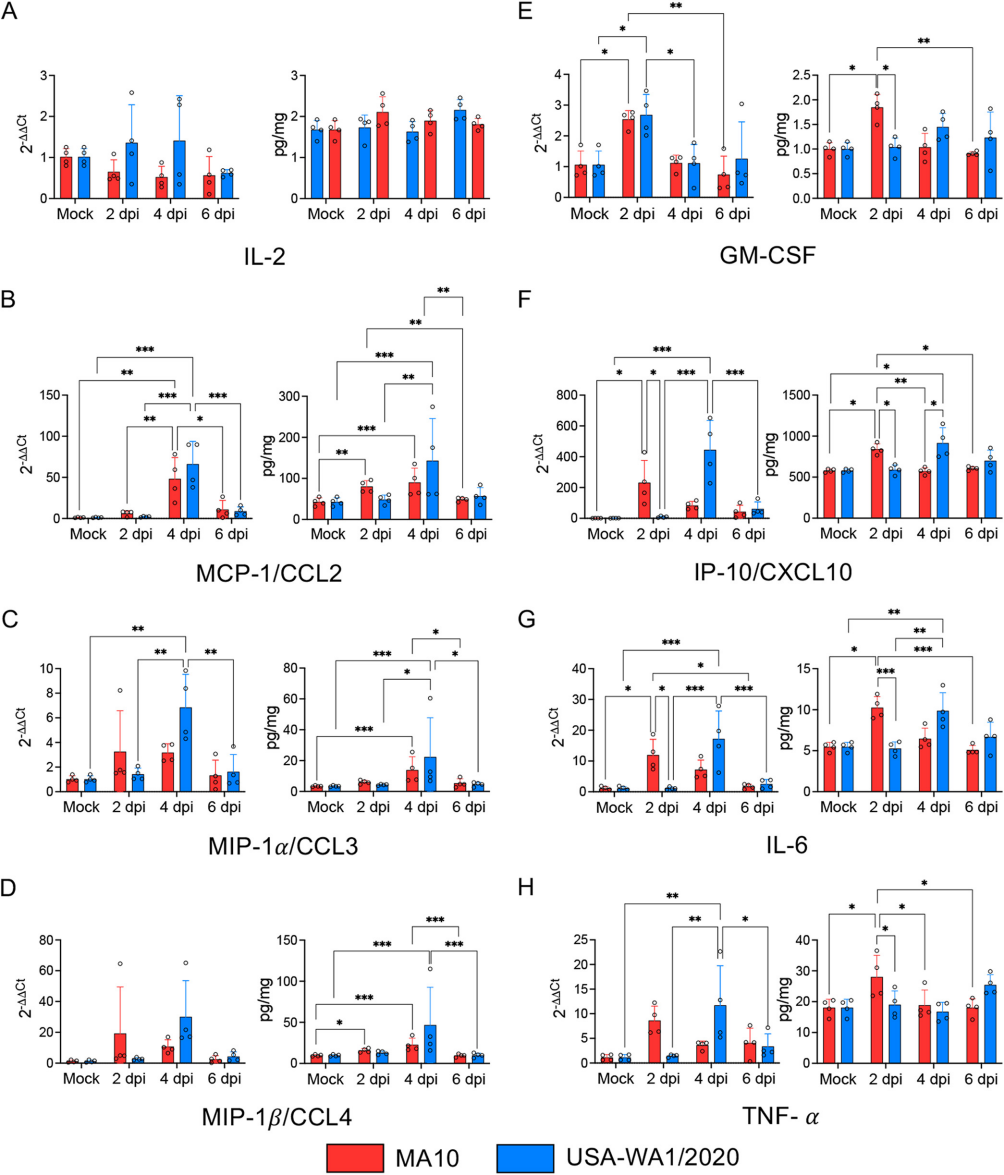
Cytokine and chemokine dynamics (protein and gene expression) in the lungs of MA10- and USA-WA1/2020-infected K18-hACE2 mice. The proinflammatory cytokine and chemokine response occurs earlier in the course of infection in the MA10-infected group compared to the parental strain. Relative gene expression and protein expression of IL-2 (A), MCP-1/CCL2 (B), MIP-1α/CCL3 (C), MIP-1β/CCL4 (D), GM-CSF (E), IP-10/CXCL10 (F), IL-6 (G), and TNF-α (H) were evaluated in the lungs of mock-, MA10-, and WA1-infected mice at 2, 4, and 6 dpi. Bars represent the mean ± standard deviation. *, *P *≤* *0.05; **, *P *≤* *0.01; ***, *P *≤* *0.001.

Gene expression of a subset of eight cytokines and chemokines reported to be related to SARS-CoV-2 pathogenesis in human patients (19, 40) was also evaluated to correlate with protein expression results. Results were consistent with protein measurements (Fig. 7). No change in IL-2 expression was observed (Fig. 7A). Overexpression of MCP-1/CCL2 (Fig. 7B), MIP-1α/CCL3 (Fig. 7C), MIP-1β/CCL4 (Fig. 7D), and GM-CSF (Fig. 7E) was noted, all of which are associated with monocyte/macrophage recruitment and responses. Interestingly, IP-10/CXCL10, IL-6 and TNF-α showed a delayed upregulation in the lung of USA-WA1/2020-infected K18-hACE2 mice (Fig. 7F to H). Overall, these data show that both strains induced an increase of proinflammatory cytokines/chemokines involved in macrophage recruitment, which are temporally delayed in the lung of USA-WA1/2020-infected K18-hACE2 mice compared to those in the MA10-infected animals.

The cytokine expression levels were also evaluated in the lung of MA10-infected C57BL/6J and BALB/c mice. In infected C57BL/6J mice, cytokine and chemokine fluctuations were mostly marginal. Among the relevant upregulated cytokines, chemokines of chemoattractant nature to monocytes were identified (i.e., MCP-5/CCL12, MIP-1α/CCL3, MIP-1β/CCL4) (see Fig. S2 in the supplemental material). These results could be associated with the overall lower susceptibility of this mouse strain to SARS-CoV-2, but also the small sample size could have contributed. Among the 10 overexpressed cytokines/chemokines detected in the infected BALB/c mice (see Fig. S3 in the supplemental material), seven of them were similar to those upregulated in the lung of MA10-infected K18-hACE2 mice (BCA-1/CXCL13, IL-6, IL-10/CXCL10, MCP-5/CCL12, MDC/CCL22, RANTES/CCL5, and SCYB16/CXCL16).

10.1128/msphere.00558-22.2 FIG S2 Expression of proinflammatory cytokines and chemokines in the lungs of MA10-infected C57BL/6J mice. Protein expression of 31 cytokines/chemokines was measured in the lung of mock- and MA10-infected C57BL/6J mice at 2 dpi, 4 dpi, and 7 dpi (*n* = 3). Data analyzed by nonparametric Wilcoxon tests. Download FIG S2, TIF file, 0.3 MB. Copyright © 2023 Thieulent et al. 2023 Thieulent et al. https://creativecommons.org/licenses/by/4.0/ This content is distributed under the terms of the Creative Commons Attribution 4.0 International license.

10.1128/msphere.00558-22.3 FIG S3 Expression of proinflammatory cytokines and chemokines in the lungs of MA10-infected BALB/c mice. Protein expression of 31 cytokines/chemokines was measured in the lung of mock- and MA10-infected BALB/c mice at 2 dpi, 4 dpi, and 7 dpi (*n* = 4). Data analyzed by nonparametric Wilcoxon tests. Download FIG S3, TIF file, 0.4 MB. Copyright © 2023 Thieulent et al. 2023 Thieulent et al. https://creativecommons.org/licenses/by/4.0/ This content is distributed under the terms of the Creative Commons Attribution 4.0 International license.

### SARS-CoV-2 induces downregulation of mouse *Ace2* expression but not human ACE2 in the lungs of K18-hACE2 mice.

The expression of human *ACE2* (*hACE2*), mouse *Ace2* (*mAce2*), and mouse *Tmprss2* (*mTmprss2*) were evaluated in the lung of infected mice. The specificity (inclusivity/exclusivity) of the primers targeting *hACE2* and *mAce2* was first validated using mRNA derived from the lungs of uninfected C57BL/6J mice, which do not express *hACE2*, and mRNA isolated from human Caco-2 cells, known to express *hACE2* (41–45) (see Fig. S4A and B in the supplemental material). Additionally, both primer sets targeting *mAce2* and *hACE2* successfully amplified *mAce2* and *hACE2* transcripts from the lung of K18-hACE2 mice (Fig. S4C).

10.1128/msphere.00558-22.4 FIG S4 Validation of the specificity of the *mAce2* and *hACE2* primer pairs. Primers used for *mAce2* and *hACE2* expression measurements were tested in mRNA extracted from lung lysates of C57BL/6J mice (A), in mRNA derived from human colorectal adenocarcinoma (Caco-2) cell pellets (B) and in mRNA extracted from lungs lysates of K18-hACE2 mice (C). Only *mAce2* or *hACE2* transcripts are detected in the lung of C57BL/6J mice or Caco-2 cells, respectively (A and B), while both *mAce2* and *hACE2* transcripts are expressed in the lung of K18-hACE2 mice (C). Δ*C_T_* values represent the difference of *C_T_* between the gene of interest and housekeeping genes. N.D., not detected. Download FIG S4, TIF file, 0.1 MB. Copyright © 2023 Thieulent et al. 2023 Thieulent et al. https://creativecommons.org/licenses/by/4.0/ This content is distributed under the terms of the Creative Commons Attribution 4.0 International license.

**FIG 8:**
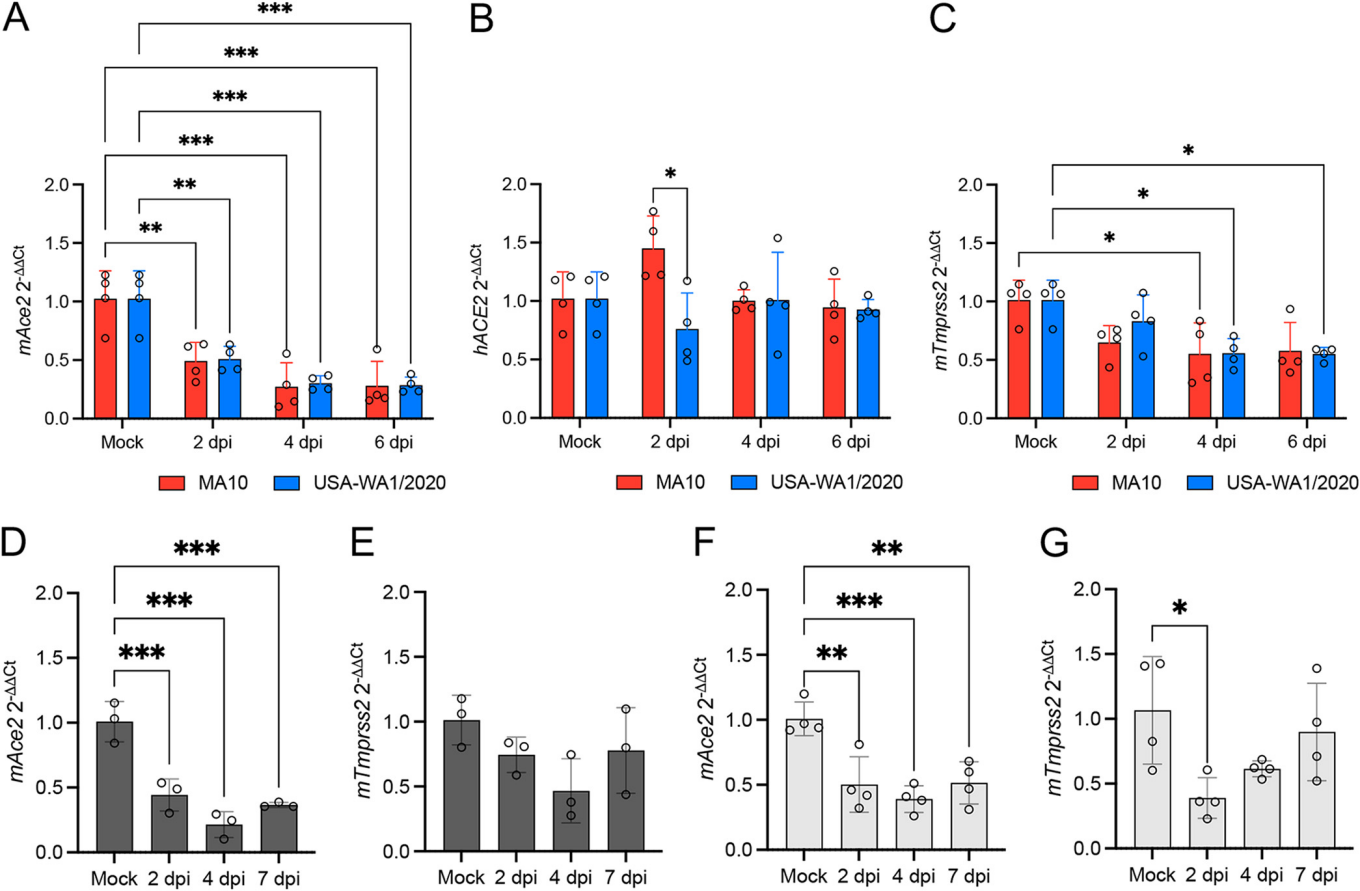
Mouse *Ace2 (mAce2)* expression, but not *hACE2*, is downregulated in the lung of SARS-CoV-2-infected K18-hACE2, C57BL/6J, and BABL/c mice. The relative expression of *mAce2* (A), *hACE2* (B), and *mTmprss2* (C) was evaluated in the lung of K18-hACE2 mice at 2, 4, and 6 dpi following infection with SARS-CoV-2 MA10 and USA-WA1/2020 strains. The relative expression of *mAce2* (D and F) and *mTmprss2* (E and G) expression were evaluated at 2, 4, and 7 dpi in the lung of mock and MA10-infected C57BL/6J and BALB/c mice, respectively. Bars represent the mean ± standard deviation. *, *P *≤ 0.05; **, *P *≤ 0.01; ***, *P* ≤* *0.001.

A significant reduction of *mAce2* expression was observed at 2 dpi in the lung of MA10- (*P = *0.005) and USA-WA1/2020-infected (*P = *0.007) K18-hACE2 mice and remained low over the course of infection (*P *≤* *0.001 for MA10- and USA-WA1/2020-infected mice) (Fig. 8A). No change in relative *hACE2* expression was observed at any stage of infection compared to that of the mock-infected mice (Fig. 8B). The relative *mTmprss2* expression was significantly reduced at 4 dpi in the lung of MA10-infected K18-hACE2 mice (*P = *0.034) and USA-WA1/2020-infected mice at 4 dpi (*P = *0.037) and 6 dpi (*P = *0.034) (Fig. 8C).

To evaluate if the downregulation of *mAce2* and *mTmprss2* occurs specifically in the lung of infected K18-hACE2 mice, we also determined the expression levels in the lungs of infected C57BL/6J and BALB/c mice. A similar trend in *mAce2* downregulation was noted in the lung of infected C57BL/6J (*P *≤* *0.001) (Fig. 8D) and BALB/c mice (*P = *0.002) (Fig. 8F). As anticipated, no *hACE2* expression was detected in either C57BL/6J or BALB/c mice (data not shown). A significant downregulation of *mTmprss2* expression was observed at 2 dpi in the lung of BALB/c mice (*P = *0.017) (Fig. 8G) but not in C57BL/6J mice (Fig. 8E).

**FIG 9:**
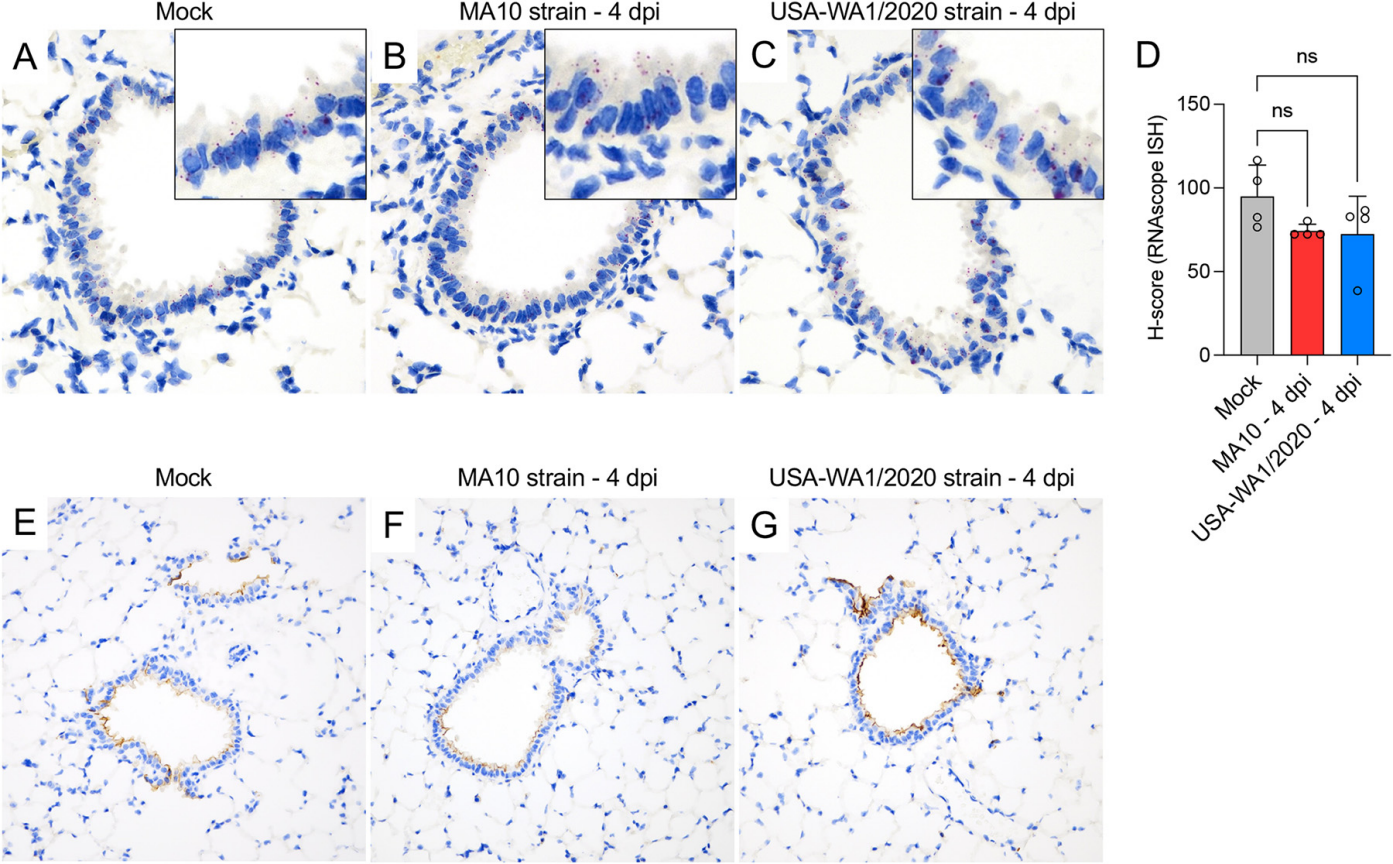
Expression and distribution of *mAce2* mRNA and ACE2 protein in the lungs of K18-hACE2 mice. *mAce2* mRNA expression and distribution was evaluated by RNAscope ISH in the lung of mock-, MA10-, and USA-WA1/2020-infected mice at 4 dpi (A to C). Expression was predominantly observed within the bronchiolar epithelium, with expression in only rare pneumocytes (A to C, insets). *mAce2* mRNA expression was semiquantitated in the bronchiolar epithelium by H-score calculation; while a reduction trend in expression level was noted, this was not statistically significant (D). Distribution of ACE2 protein in the lung of mock-, MA10-, and USA-WA1/2020-infected mice at 4 dpi (E to G). ACE2 has an apical distribution predominantly within bronchiolar epithelium and rarely in AT2 cells. Fast Red (*mAce2* mRNA) (A to C) and DAB (brown, ACE2) (E to G). ×200 total magnification.

The *mAce2* expression and distribution was also analyzed by RNAscope *in situ* hybridization (ISH) using a mouse-specific probe and by IHC in K18-hACE2 mice (Fig. 9). Expression of *mAce2*/ACE2 mainly involved bronchiolar epithelial cells with apical protein expression, and sporadic expression in AT2 cells (Fig. 9A to C and Fig. 9E to G). Tissue-based quantification of *mAce2* noted a reduction trend of *mAce2* expression at 4 dpi in both infected groups; statistical significance was not reached as observed via quantitative PCR (qPCR) analysis, likely due to differences in assay’s sensitivity (Fig. 9D, MA10 versus Mock, *P = *0.078; USA-WA1/2020 versus Mock, *P = *0.753). No differences in the distribution and abundance of ACE2 expression were identified between mock-infected and both infected groups at 4 dpi (Fig. 9E to G).

## DISCUSSION

Since the discovery of SARS-CoV-2, several animal models have been used to understand its pathophysiology (46–48). Based on the low affinity interaction between murine ACE2 and the RBD of the ancestral, Wuhan-like SARS-CoV-2 S glycoprotein, the transgenic mouse expressing hACE2 under the K18 promoter (K18-hACE2) has become, among others, an extensively used model to study SARS-CoV-2 pathogenesis and preclinically assess therapeutic and vaccine efficacy (29). The Syrian hamster is another popular model for such studies with some advantageous features compared to the K18-hACE2 model, including clinical recovery following infection (i.e., nonlethal model), lack of neuroinvasion/neurodissemination, and development of pronounced bronchointerstitial pneumonia with subsequent intense airway reparative response. Such features have allowed better parallelism with disease in humans (49–51). However, not all animal biosafety level 3 (ABSL-3) facilities have the necessary infrastructure to handle this rodent model and also the lack of immunological reagents available limits their utilization (52). Currently, there is no animal model that perfectly recapitulates all of the features of COVID-19, and, therefore, careful selection and continued refinement of appropriate rodent models to test hypotheses, vaccine efficacy, and treatments related to understanding the mechanisms of SARS-CoV-2 in humans is extremely important. As an alternative approach, Leist et al. developed a mouse-adapted SARS-CoV-2 strain (namely, MA10) engineered to bind mACE2 and to which conventional mouse strains are permissive (34). To date, the vast majority of the studies in the literature have evaluated the pathogenicity of Wuhan-like SARS-CoV-2 and VOCs (i.e., Alpha, Beta, Delta, and Omicron variants) in K18-hACE2 mice (35, 53–55), with only a single study evaluating the replication of the mouse-adapted MA10 strain in this specific mouse model (39). Thus, far, no in-depth characterization of the differences in infection dynamics and pathophysiology between the mouse-adapted MA10 strain and the counterpart USA-WA1/2020 in K18-hACE2 mice has been undertaken. Therefore, the main objective of this study was to compare the dynamics and differential pathogenesis of both MA10 and its parental strain, USA-WA1/2020, on the widely used K18-hACE2 mouse model. Overall, our study demonstrated that the mouse-adapted MA10 strain induces earlier clinical decline, accelerated viral replication, earlier shift in pulmonary host responses, and displays differential tropism compared to its parental counterpart in K18-hACE2 mice. This comparison is highly relevant, as it provides critical comparative data on viral tropism and temporal dynamics that would better inform study design when utilizing these strains in this widely used mouse model.

Based on the analysis of lungs from autopsied patients, SARS-CoV-2 antigen is mainly detected in the majority of bronchiolar epithelial cell types and occasional AT1 and AT2 cells (56–58). In this study, we demonstrated that the MA10 strain can infect both bronchiolar and alveolar (AT1 and AT2) epithelia in K18-hACE2 mice, whereas the tropism of the USA-WA1/2020 strain is restricted only to alveolar (AT1 and AT2) cells. Since differences in tropism could be related to cell-specific expression of ACE2, the main host receptor for SARS-CoV-2, we sought to analyze its expression in the lung of mice. We demonstrated that *mAce2* is mainly expressed in the bronchiolar epithelium and in scattered AT2 cells (59). Similar localization of *hACE2*, when driven by the K18 promoter, was recently demonstrated (35), but surprisingly, no viral antigen was detected within bronchiolar epithelia after USA-WA1/2020 infection. Interestingly, when comparing viral tropism of the MA10 strain in alveolar cells of K18-hACE2 and BALB/c mice, we observed that, while viral antigen is diffusely expressed in both AT1 and AT2 cells in K18-hACE2, viral antigen seems mostly restricted to AT2 cells in BALB/c mice. Results from our study suggest that the MA10 strain exploits both mACE2 and hACE2 receptor, and utilization of mACE2 is likely responsible for the increased cellular tropism and infection of bronchiolar epithelium. Such observation is also supported by the earlier neuroinvasion noted in MA10-infected K18-hACE2 mice. While the bronchiolar epithelial infection is transient, the use of the MA10 strain in the K18-hACE2 mice seems to be an alternative model with a more comparable tropism to that seen in COVID-19 patients than that of the USA-WA1/2020 strain.

Interestingly, our results demonstrate a pronounced and specific downregulation of *mAce2* expression after SARS-CoV-2 infection, in the absence of similar changes in *hACE2* transcripts, regardless of the virus strain used. This is in contrast to a previous study (29), where *hACE2* expression was reported to be decreased in the lung of K18-hACE2 mice after infection with a similar dose of USA-WA1/2020. The *hACE2* expression in this previous study was measured in copy per mg of tissue (i.e., absolute quantification), which cannot be compared head-to-head with relative quantification (29). However, the specificity of the primer-probe combination used and the absence of *mAce2* amplification were not reported. The specificity of our assays was thoroughly evaluated in wild-type and *hACE2* transgenic mice, and the Caco-2 human-derived cell line known for expressing *ACE*2 (41–45). The reduction of *mAce2* expression was also observed in the lung of infected C57BL/6J and BALB/c mice, confirming that it is indeed *mAce2*, not *hACE2*, that is downregulated in the lung of infected K18-hACE2 mice. The inability to detect a discernible decline of ACE2 protein expression by IHC is explained by the cross-reactive nature of the anti-ACE2 antibody used for both hACE2 and mACE2. Unfortunately, there were no monospecific antisera to either hACE2 or mACE2 to make a clear distinction between the expression of these two receptors in mouse tissues using IHC. It was previously demonstrated that *mAce2* expression is downregulated in the lung of mice infected with SARS-CoV by Western blotting (60). Similar results were obtained with influenza A H1N1 infections (61) and influenza A H5N1 (62). The lack of downregulation of *hACE2* could possibly be associated with the high number of transgene copies in K18-hACE2 mice as previously reported (63). Further studies are needed to understand the pathogenic relevance of this change and its potential impact on pulmonary responses. To our knowledge, no previous study reports a change in *Tmprss2* expression after SARS-CoV-2 infection. Here, we identified a reduction of *Tmprss2* expression in the lung of K18-hACE2 mice regardless of the SARS-CoV-2 strain used and a transient reduction in the lung of BALB/c mice infected with the MA10 strain. Interestingly, only a trend of *Tmprss2* downregulation was observed in the lungs of infected C57BL/6J mice, which is the least susceptible mouse strain to MA10 infection (34). Epithelial turnover after SARS-CoV-2 infection may be an explanation for the transient *Tmprss2* downregulation, since *Tmprss2* is rich within bronchiolar epithelial cells (see Fig. S5 in the supplemental material).

10.1128/msphere.00558-22.5 FIG S5 Expression of *mTmprss2* mRNA in the lung of mice using RNAscope ISH. *Tmprss2* is rich within bronchiolar epithelia (inset 1) and sporadic in AT1 and AT2 cells (inset 2). Fast Red (*mTmprss2* mRNA); ×200 total magnification. Download FIG S5, TIF file, 3.9 MB. Copyright © 2023 Thieulent et al. 2023 Thieulent et al. https://creativecommons.org/licenses/by/4.0/ This content is distributed under the terms of the Creative Commons Attribution 4.0 International license.

An additional important feature of MA10 infection in K18-hACE2 mice is related to the earlier pulmonary innate immune responses and differences in the cytokine/chemokine inflammatory signature compared to the parental strain. This, along with the overexpression of a higher number of proinflammatory cytokines/chemokines in the lung of MA10-infected mice, is related to the accelerated viral replication with rapid increase in viral titers and with the differential pulmonary tropism compared to its parental strain. The lack of IL-2 overexpression, coupled with the increase of cytokines/chemokines involved in macrophage and monocyte recruitment (i.e., IL-6, MCP-1/CCL2, and GM-CSF) reflects the fact that the local cellular response in the infected lung is mediated primarily by macrophages rather than lymphocytes (35). Of note, the absence of a pronounced IFN-α-mediated response in the lung of K18-hACE2 mice after infection with both strains suggests inhibition of type I interferons, which is consistent with previous reports in infected human patients (64).

The lack of SARS-CoV-2 neuroinvasion in conventional laboratory mice (i.e., BALB/c and C57BL/6J) showed in this as well as other studies (31, 65, 66) further highlights that hACE2 is a key but likely not the sole factor associated with the neuropathogenesis in the K18-hACE2 model (35). As previously reported, lethality in this mouse model is associated with viral neuroinvasion and neurodissemination via transport to the olfactory bulb originating from axonal processes traversing the olfactory neuroepithelium (35). Based on the rapid neurodissemination following intranasal challenge with the MA10 strain, we hypothesize that the presence of both mACE2 and hACE2 facilitates entry and rapid dissemination, explaining the higher level of viral titers/viral RNA and antigen in the brain by 4 dpi and the earlier weight loss. This difference occurs within a narrow time frame following infection as both MA10- and USA-WA1/2020-infected K18-hACE2 mice reach similar levels of viral replication, antigen abundance, histologic alterations, and clinical decline/mortality by 6 dpi.

Based on all of these observations, the use of the MA10 strain in the K18-hACE2 model provides a better representation of human COVID-19 in terms of viral tropism and progression of bronchointerstitial pneumonia, yet it is still imperfect and with important limitations that overall preclude the mouse from being the most faithful or relevant model of COVID-19. Major limitations in the utilization of both wild-type and K18-hACE2 mice have been identified (here and elsewhere) and mainly include marginal disease phenotypes (in wild-type mice) and absence of interstitial pneumonia uniquely depicting the features of diffuse alveolar damage (DAD) and subsequent reparative fibrosis that characterize pneumonia in human patients (absent in both wild-type and transgenic mice). Additionally, SARS-CoV-2 neuroinvasion and neurodissemination specifically makes the K18-hACE2 model invariably and prematurely fatal and, thus, limits its use for studying virus-specific immune responses, pulmonary repair mechanisms, and viral long-term effects (i.e., post-acute sequela of COVID-19 [long COVID-19]). Another important consideration relates to the virus strain used, and the utilization of the mouse-adapted MA10 strain may not necessarily reflect the pathogenicity of VOCs currently circulating in the population. Therefore, the mouse model cannot be appraised as the sole model for comprehensive understanding of SARS-CoV-2 pathogenesis, and multiple alternative animal models are needed to understand different aspects of COVID-19 in a complementary manner. Consequently, thorough characterization of virus strains in specific animal models and identification of their limitations is necessary to assess translational potential and further refine model systems.

To conclude, this study provides further insight into the comparative dynamics of SARS-CoV-2 MA10 and USA-WA1/2020 strains in the K18-hACE2 transgenic mouse model along with temporal pulmonary *mAce2*, *hACE2*, and *mTmprss2* expression analysis. This study provides extensive evidence that the MA10 strain replicates and disseminates more rapidly in the lungs and brain of K18-hACE2, exhibits differences in pulmonary tropism, and induces an earlier proinflammatory and monocyte chemoattractant chemokine response compared to the parental USA-WA1/2020 strain; usage of both mACE2 and hACE2 as host receptors by MA10 is likely the underlying mechanism driving these dynamics. These differences could be of significant impact to experimental conditions, and, therefore, this study provides critical information that should be considered for study design based on strain selection, strain differences in cell tropism, and the experimental time points selected for downstream analysis. Using the MA10 strain along with the K18-hACE2 mouse model represents an additional model for understanding the mechanisms governing SARS-CoV-2 pathogenesis and evaluating the efficacy of therapeutics and vaccines.

## MATERIALS AND METHODS

### Biosafety.

All aspects of this study were approved by the Institutional Biosafety and Recombinant DNA Safety Committee, Office of Environmental Health and Safety at Louisiana State University (LSU) prior to study initiation. Work with SARS-CoV-2 was performed in a biosafety level-3 (BSL-3 and ABSL-3) laboratory at the School of Veterinary Medicine, LSU by personnel equipped with powered air-purifying respirators.

### Ethical statement.

All experimental procedures were approved by the Institutional Animal Care and Use Committee at Louisiana State University (IACUC protocol number 20-091). Euthanasia was performed via isoflurane overdose and subsequent cervical dislocation following the American Veterinary Medical Association (AVMA) Guidelines for the Euthanasia of Animals.

### Cells and viruses.

African green monkey VERO C1008 cells (Vero 76, clone E6, Vero E6) (ATCC CRL-1587, Manassas, VA) were maintained in minimum essential medium (MEM) with Earl’s salts and l-glutamine (Corning, Corning, NY) supplemented with 10% fetal bovine serum (FBS) (HyClone Laboratories, Inc., Logan, UT), 100 U/mL of penicillin and 100 μg/mL streptomycin (Gibco, Carlsbad, CA), and 0.25 μg/mL of amphotericin B (Gibco). Caco-2 colorectal adenocarcinoma cells (ATCC HTB-37) were maintained in MEM as described above but contained 20% FBS. Cells were incubated at 37°C and 5% CO_2_ in a humidified incubator.

The SARS-CoV-2 mouse-adapted strain, MA10 variant (NR-55329), and the SARS-CoV-2 isolate USA-WA1/2020 (NR-52281) were obtained from BEI Resources (Manassas, VA, USA). Virus stocks (passage 3) were prepared in Vero E6 cells, titrated by plaque assay, and stored at −80°C for experimental infections. The genomes of both SARS-CoV-2 MA10 and USA-WA1/2020 strains at passage 3 were fully sequenced at the Iowa State University Veterinary Diagnostic Laboratory, Ames, IA. Consensus genome sequences were analyzed using Geneious R7 (BioMatters, Auckland, New Zealand) and demonstrated to possess 99.9% and 100% homology with the reference genome sequences, respectively (MA10 strain [GenBank accession number MT952602.1]; USA-WA1/2020 strain [GenBank accession number MW811435.1]).

### Mice.

Twelve-week-old male B6.Cg-Tg(K18-ACE2)2Prlmn/J (strain number 034860, K18-hACE2; *n* = 28) and 14-week-old male C57BL/6J mice (strain no. 000664; *n* = 12) were obtained from The Jackson Laboratory (JAX, Bar Harbor, ME). Additionally, 10-week-old male BALB/cAnNHsd mice (strain number 047, BALB/c; *n* = 16) were obtained from Envigo (Indianapolis, IN). Mice were housed in groups of 3 to 4 per cage under a 12-h light/12-h dark cycle with *ad libitum* access to water and a standard chow diet.

### Experimental design and intranasal inoculation.

Since K18-hACE2 mice typically reach humane euthanasia criteria (associated with viral neurodissemination) within the first week following SARS-CoV-2 infection (28, 29, 35, 67), all experiments were performed for a maximum duration of 7 dpi. K18-hACE2 mice were randomly assigned to the MA10-infected group (*n* = 12), WA1-infected group (*n* = 12), or the mock-infected group (*n* = 4). C57BL/6J and BALB/c mice were randomly assigned to the MA10-infected group (*n* = 9 and *n* = 12, respectively) or the mock-infected group (*n* = 3 and *n* = 4, respectively). For intranasal inoculation, mice were anesthetized with 4% isoflurane (Fluriso; VetOne, Boise, ID) (oxygen, 3 to 4 L/min) in an induction chamber until deeply anesthetized. Mice were intranasally inoculated with either 50 μL (25 μL per nostril) of MEM containing 2 × 10^4^ PFU of the SARS-CoV-2 MA10 strain, SARS-CoV-2 USA-WA1/2020 strain, or mock inoculated with 50 μL of plain MEM. Mice were then transferred to their cages and monitored until completely recovered (approximately 10 min). Three (C57BL/6J) and four (K18-hACE2 and BALB/c) infected mice per group were euthanized at 2 dpi, 4 dpi, and 7 dpi or when mice reached predetermined humane euthanasia criteria. All mock-infected mice were euthanized at 6 dpi (K18-hACE2) or 7 dpi (C57BL/6J and BALB/c).

### Clinical monitoring.

An IACUC-approved clinical scoring system was utilized to monitor disease progression as previously described (35). Clinical signs and body weight were monitored once a day for the duration of the experiment (7 dpi). A score of 0 or 1 was given for each of the following parameters: body weight loss greater than 15%; rapid/shallow respiration or increased respiratory effort; appearance (ruffled fur, hunched posture); responsiveness; and neurologic signs (e.g., tremors). Cumulative scores of 4 or greater for two consecutive observation periods, weight loss greater than or equal to 15%, severe respiratory distress, or lack of responsiveness were the criteria considered for electing humane euthanasia.

### Tissue collection and processing.

The main left bronchus was clamped using Kelly forceps, and the right lung was insufflated with instillation of ~750 μL of 1% low-melting-point agarose (Thermo Fisher Scientific, Waltham, MA) diluted in 1× phosphate-buffered saline using a 24-gauge catheter placed into the trachea. The left lung was divided into ~30 mg samples and stored at −80°C either as fresh frozen or in RNAlater solution (Invitrogen, Waltham, MA) in 1.5 mL microfuge tubes containing 2.4 mm metal grinding beads (Omni International, Kennesaw, GA). Subsequently, the skull cap was removed, and ~30 mg of left frontal cortex was collected and stored at −80°C in 1.5 mL microfuge tubes containing 2.4 mm metal grinding beads. The insufflated right lung and head were fixed in 10% neutral buffered formalin at a 20:1 fixative to tissue ratio for a minimum of 72 h before removal from BSL-3 in accordance with an approved institutional standard operating procedure.

### Viral RNA isolation and quantitation by SARS-CoV-2 ORF1ab-specific real-time RT-qPCR.

Approximately 30 mg of fresh frozen lung and brain were homogenized using a Qiagen TissueLyser II (Qiagen, Valencia, CA) in MEM (1:10 volume; 10% tissue homogenates) by two dissociation cycles of 2 min at 30 Hz. Samples were clarified by centrifugation at 3,000 × *g* for 10 min at 4°C. A total of 100 μL of clarified 10% tissue homogenate were collected, and viral RNA isolation was subsequently performed using the Taco nucleic acid automatic extraction system (GeneReach Biotechnology Corporation, Taichung, Taiwan) as previously described (68) and eluted in 100 μL of elution buffer. Reverse transcription-quantitative PCR (RT-qPCR) targeting ORF1ab of SARS-CoV-2 (69, 70) was performed using a 7500 fast real-time PCR system (Applied Biosystems). Sequences of primers, probe, and thermal cycling are shown in Table 1. RT-qPCRs were performed in a total volume of 20 μL containing 5 μL of 4× TaqPath 1-step RT-qPCR master mix (Applied Biosystems), 1 μL of 20× primers (500 nM)/probe (250 nM) mix, 6 μL of nuclease-free water, and 5 μL of extracted RNA. For absolute quantitation of viral RNA, a standard curve using a synthesized *in vitro* transcribed RNA containing a 132-bp fragment from the SARS-CoV-2 ORF1ab gene was used. Briefly, the target sequence was chemically synthesized and cloned into the pGEM-3Z vector (Promega, Madison, WI), and *in vitro* RNA transcription was performed as previously described (71, 72). Synthesized RNA was purified using the MEGAclear transcription clean-up kit (Thermo Fisher Scientific) and quantitated using a Qubit 4 fluorometer (Invitrogen). The analytical sensitivity of the assay was determined to be 1 genome copy/μL.

**TABLE 1:** Primers and probe sequences used for SARS-CoV-2 RNA quantitation

Target	Primers and probe sequences (5′–3′)^*a*^	Cycling temp and time	Reference
SARS-CoV-2	HKU-ORF1-F: TGGGGYTTTACRGGTAACCT	25°C, 2 min	69
ORF1ab	HKU-ORF1-R: AACRCGCTTAACAAAGCACTC	50°C, 15 min	
	HKU-ORF1-P: FAM-TAGTTGTGATGCWATCATGACTAG-BHQ1	95°C, 2 min^*b*^	
		95°C, 3 sec^*b*^	
		60°C, 30 sec^*b*^	

a F, forward; R, reverse; P, probe.

b 40 cycles of amplification.

### Virus titer determination by plaque assay.

Clarified (10%) lung and brain homogenates were subjected to 10-fold serial dilutions in MEM and inoculated in Vero E6 cells preseeded in 12-well plates. After 1 h of adsorption at 37°C, cells were overlaid with complete MEM containing 0.75% carboxymethylcellulose (Sigma-Aldrich, Saint-Louis, MO) and incubated for 72 h at 37°C and 5% CO_2_ in a humidified incubator. Media was finally removed, and cells were fixed and stained with 0.2% crystal violet solution (Sigma-Aldrich) in 10% neutral buffered formalin for 4 h and subsequently rinsed with tap water. Plaques were counted, and viral titers were determined as PFU per milligram of tissue.

### Chemokine and cytokine analysis.

Chemokine and cytokine protein expression analysis was performed on collected lung tissue using the Bio-Plex Pro mouse chemokine panel 31-plex (Bio-Rad, Hercules, CA). Approximately 30 mg of lung was homogenized in 500 μL of Bio-Plex cell lysing solution (Bio-Rad) according to the manufacturer’s recommendations using a Qiagen TissueLyser II as described above, followed by one freeze-thaw cycle at −80°C. Samples were centrifuged at 4,500 × *g* for 10 min at 4°C and subsequently diluted 1:10 in sample diluent, processed following the manufacturer’s recommendations, and read on a Bio-Plex 200 system (Bio-Rad). Each sample was analyzed in duplicate wells. The concentration of interferon-alpha (IFN-α) was measured using the mouse IFN-α enzyme-linked immunosorbent assay (ELISA) kit (Invitrogen) according to the manufacturer’s recommendations. Each sample was analyzed in duplicate wells, and absorbance was read at 450 nm using a BOLT automated ELISA system (Gold Standard Diagnostics, Davis, CA). Cytokine/chemokine concentrations were calculated as picograms per milligram of tissue.

### Total cellular RNA isolation and gene expression analysis for selected cytokines/chemokines.

Gene expression analysis of cellular genes involved in SARS-CoV-2 binding/entry (*mAce2*, *hACE2*, and *mTmprss2*) as well as a subset of cytokines/chemokines, including *Mcp1/Ccl2*, *Mip1a/Ccl3*, *Mip1b/Ccl4*, *Gm-csf*, *Il2*, *Il6*, *Ip10/Cxcl10*, and *Tnfa*, were measured as previously described (73). Approximately 30 mg of frozen lung tissue was homogenized in 600 μL of Buffer RLT Plus (Qiagen) containing 1% of β-mercaptoethanol using a Qiagen TissueLyser II (Qiagen) as described above. Total RNA was extracted using the RNeasy Plus minikit (Qiagen) according to the manufacturer’s recommendations, and then 2 μg of total RNA was reverse transcribed using the high-capacity cDNA reverse transcription kit (Applied Biosystems) according to the manufacturer’s recommendations. The 2× PowerUp SYBR green master mix (Applied Biosystems) was used for qPCRs, and these were performed using a QuantStudio 5 real-time PCR system (Applied Biosystems) as previously described (73). Melt curve analysis was performed to confirm nonspecific amplifications along with the inclusion of nontemplate controls. Specific forward and reverse primers for genes of interest were designed using the Primer-BLAST website (NCBI, NIH) and are shown in Table 2. Specificity of the *mAce2* and *hACE2* primers were confirmed using RNA extracted from lungs of C57BL/6J mice and Caco-2 (Caco2) (ATCC HTB-37) cell pellets. Quantitative PCR efficiencies and cycle threshold (*C_T_*) values were determined using LinRegPCR version 2021.2 (74). Reactions were performed in duplicate and expression of genes of interest were normalized to three stable housekeeping genes (*Actb*, *Gusb*, and *Rpl13b*) as previously described (75).

**TABLE 2:** Primer sequences used for gene expression analysis

Target (GenBank accession no.)^*a*^	Forward primer (5′–3′)	Reverse primer (5′–3′)	Product length (bp)
Human *ACE2* (NM_001371415.1)	AGAAAGCAGTCTGCCATCCC	GCTGTCAGGAAGTCGTCCAT	97
Mouse *Ace2* (NM_001130513.1)	GTCATGGATGCGCTTTGGAT	CTTGGGTTGGGCACTGCTTA	88
*Tmprss2* (NM_015775.2)	TGACGGGGTAGCACATTGTC	CACACGGGATACCAGGCTTT	117
*Ccl2* (NM_011333.3)	TGACCCCAAGAAGGAATGGG	ACCTTAGGGCAGATGCAGTT	104
*Ccl3* (NM_011337.2)	ACATTCCTGCCACCTGCATA	GAAGAGTCCCTCGATGTGGC	74
*Ccl4* (NM_013652.2)	CTAACCCCGAGCAACACCAT	CCATTGGTGCTGAGAACCCT	97
*Cxcl10* (NM_021274.2)	CTGAGTCCTCGCTCAAGTGG	GTCGCACCTCCACATAGCTT	69
*Gm-csf* (NM_009969.4)	GCCAGTTCTTGGAAGGGCTTAT	ATCTCCTGGCCCTTATCAGCTA	99
*Il-2* (NM_008366.3)	GAAACTCCCCAGGATGCTCA	CGCAGAGGTCCAAGTTCATCT	99
*Il-6* (NM_031168.2)	GGGACTGATGCTGGTGACAA	ACAGGTCTGTTGGGAGTGGT	90
*Tnfα* (NM_013693.3)	AGCCGATGGGTTGTACCTTG	ATAGCAAATCGGCTGACGGT	99
*Actb* (NM_007393.5)	CACTGTCGAGTCGCGTCC	TCATCCATGGCGAACTGGTG	89
*Gusb* (NM_001357025.1)	GGCGATGGACCCAAGATACC	ACCCTTGGGATACCACAACT	84
*Rpl13a* (NM_001357025.1)	CCCACAAGACCAAGAGAGGC	CACCATCCGCTTTTTCTTGTCA	92

a *Ace2/ACE2*, angiotensin converting enzyme 2; *Tmprss2*, transmembrane serine protease 2; *Ccl*, C-C motif chemokine ligand; *Cxcl10*, C-X-C motif chemokine ligand 10; *Gm-csf*, granulocyte-macrophage colony-stimulating factor; *Il*, interleukin; *Tnfα*, tumor necrosis factor α; *Actb*, actin, beta; *Gusb*, glucuronidase beta; *Rpl13a*, ribosomal protein L13a.

### Histopathology.

Following fixation, the whole head was decalcified in Immunocal Decalcifier (StatLab, McKinney, TX) for 5 days followed by thorough rinsing in tap water per manufacturer’s recommendations before performing a midsagittal section. Tissues (lung and heads) were subsequently processed, embedded in paraffin, and 4 μm sections stained with hematoxylin and eosin following standard histological procedures. Histological alterations were semiquantitatively scored as follows: 0, no histologic alterations; 1, minimal; 2, mild; 3, moderate; and 4, severe.

### SARS-CoV-2-specific immunohistochemistry.

For SARS-CoV-2 IHC, 4 μm sections of formalin-fixed paraffin-embedded tissue were mounted on positively charged Superfrost Plus slides and subjected to IHC using antinucleocapsid rabbit polyclonal antibody (3A) as previously described (76–82) on the automated BOND-RXm platform (Leica Biosystems, Nussloch, Germany). SARS-CoV-2 N antigen abundance was semiquantitatively scored as follows: 0, no viral protein observed; 1, up to 5% positive target cells per 400× field examined; 2, 5 to 25% positive target cells per 400× field examined; 3, up to 50% positive target cells per 400× field examined; and 4, >50% positive target cells per 400× field examined. Additionally, the immunoreactive area was quantitated as indicated below.

For ACE2, tissue sections were prepared as described above and epitope retrieval performed using a ready-to-use EDTA-based solution (pH 9.0; Leica Biosystems) at 100°C for 20 min. Sections were then incubated with a ready-to-use hydrogen peroxide solution (Leica Biosystems) for 5 min and subsequently a rabbit monoclonal anti-ACE2 antibody (clone E5O6J [Cell Signaling Technologies, Danvers, MA, USA] diluted at 1:100 in primary antibody diluent [Leica Biosystems]) for 30 min at room temperature. Slides were finally incubated with a polymer-labeled goat anti-rabbit IgG coupled with horseradish peroxidase (8 min). 3′,3′ diaminobenzidine (DAB) was used as the chromogen (10 min), and counterstaining was performed with hematoxylin for 5 min. Slides were dried and mounted as indicated above.

### RNAscope *in situ* hybridization for mouse *Ace2*.

For mouse *Ace2* mRNA RNAscope ISH, an antisense probe targeting mouse *Ace2* (GenBank accession number NM_001130513.1; catalog number 863818) with no cross-reactivity to *hACE2* was used. For mouse *Tmprss2* mRNA RNAscope ISH, an anti-sense probe targeting mouse *Tmprss2* (GenBank accession number NM_015775.2; catalog number 496728) was used. The RNAscope ISH assay was performed using the RNAscope 2.5 LSx reagent kit (Advanced Cell Diagnostics, Newark, CA) on the automated BOND RXm platform (Leica Biosystems, Buffalo Grove, IL) as described previously (35).

### Quantitative image analysis of SARS-CoV-2 antigen distribution and abundance.

Digitized whole-slide scans were analyzed using the image analysis software HALO (Indica Labs, Inc., Corrales, NM). Slides were manually annotated to include only the brain and/or lung parenchyma. Visualization threshold values were adjusted in viewer settings to reduce background signal. Area quantification (AQ) was performed to determine percentages of SARS-CoV-2 N antigen immunoreactivity in lungs and brains.

### Quantitative image analysis of mouse *Ace2* abundance.

Quantitative analysis for RNAscope ISH to mouse Ace2 was performed in QuPath 0.3.1 digital pathology image analysis software. Guidelines established by ACD were followed with some modifications. Briefly, stain vectors were adjusted for each slide, respectively, before pursuing further analysis. Since mouse *Ace2* is most abundant in the bronchiolar epithelium, five approximately 300-μm-diameter bronchioles were randomly selected for analysis. The cell detection algorithm was performed with default values, and after cell segmentation was performed, the subcellular detection algorithm was applied for detection of Fast Red (*Ace2*) spots using default values except that minimum spot size was set to 0.1 and thresholds were fine-tuned for detection accuracy. The estimated number of spots were determined per cell per region and exported into an Excel file. Spots/cell (excluding clusters) for each tissue compartment were used to compute an H-score (range of 0 to 400) by binning cells with different levels of expression into separate bins (bin 0 [0 dots/cell], bin 1 [1 to 3 dots/cell], bin 2 [4 to 9 dots/cell], bin 3 [10 to 15 dots/cell], and bin 4 [greater than or equal to 15 dots/cell]). The H-score was computed using the weighted formula as follows: H-score = ∑ (bin number × % cells per bin). H-scores were subsequently used for statistical analysis.

### Statistical analysis.

Statistical analysis was performed using GraphPad Prism v9.3.1 statistical analysis software (GraphPad, San Diego, CA) and JMP16 Pro (Cary, NC, USA). Kaplan-Meier curves were used for survival analysis. Weight changes were analyzed using two-way analysis of variance (ANOVA) with Sidak’s as the *post hoc* test. Viral loads (genomic copies/mg and PFU/mg) and relative gene expression levels were analyzed using two-way ANOVA with Tukey’s as the *post hoc* test. Clinical, histopathology, and viral antigen abundance scores and RNAscope ISH H-scores were analyzed using the nonparametric Kruskal-Wallis test followed by a Dunn’s *post hoc* test using the mock group as control. Chemokine and cytokine protein concentrations and percentages of immunoreactivity area were analyzed using the nonparametric Kruskal-Wallis test and a Dunn’s *post hoc* test for joint ranks. Fold change of cytokine and chemokine protein concentrations was calculated by dividing the protein concentrations of infected mice by the average protein concentrations of mock-infected mice, and a heatmap was build using package “pheatmap” in R. Data are expressed as mean ± standard deviation (SD). *, *P *≤* *0.05; **, *P *≤* *0.01; ***, *P *≤* *0.001.
