# Supplementary figures and images for: Mouse-Adapted SARS-CoV-2 MA10 Strain Displays Differential Pulmonary Tropism and Accelerated Viral Replication, Neurodissemination, and Pulmonary Host Responses in K18-hACE2 Mice
Source: mSphere. 2023 Feb 2;8(1):e00558-22. doi: 10.1128/msphere.00558-22 (PMC9942576; doi:10.1128/msphere.00558-22)

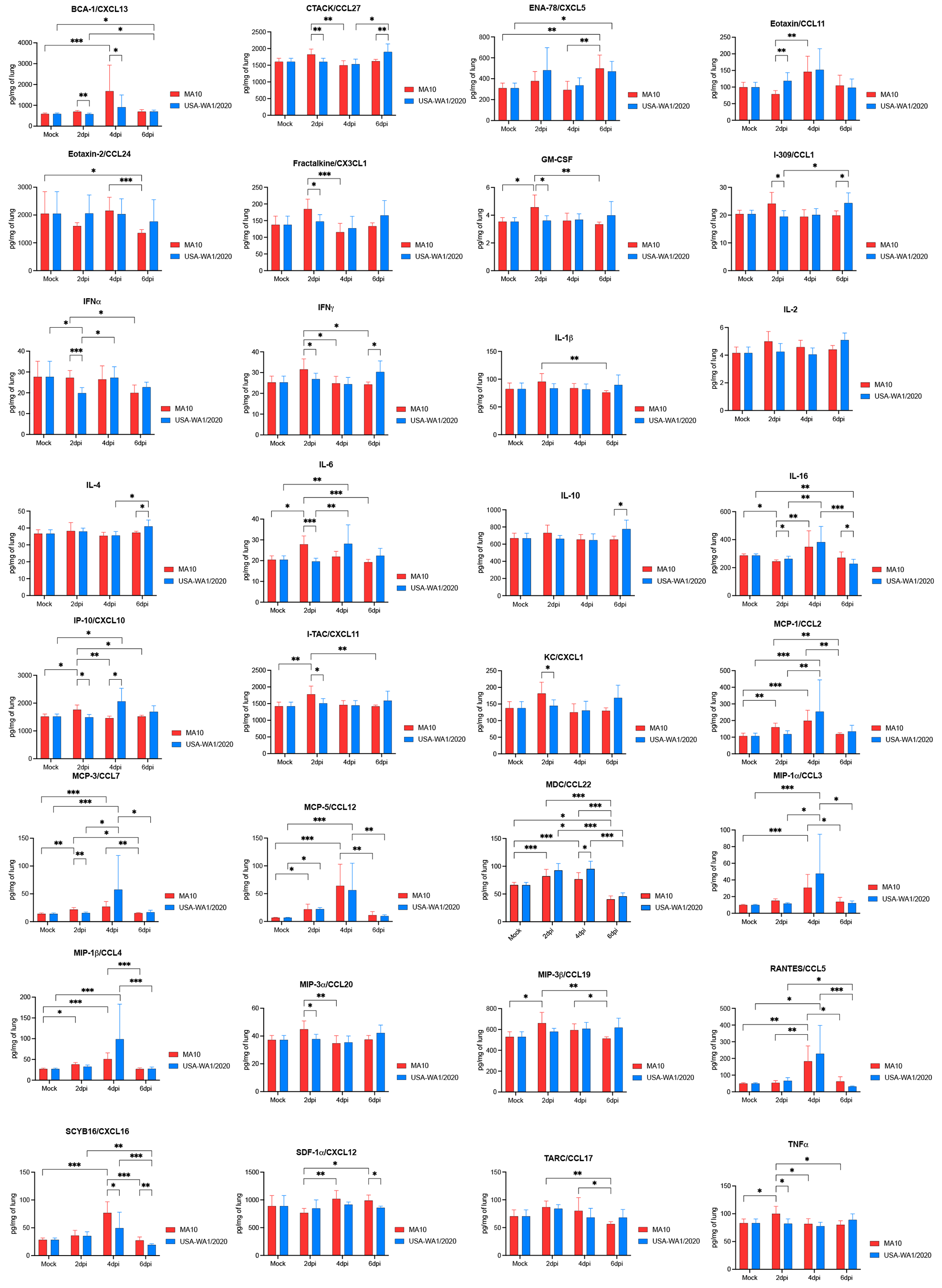

Supplement: FIG S1 [file msphere.00558-22-s0001.tif]

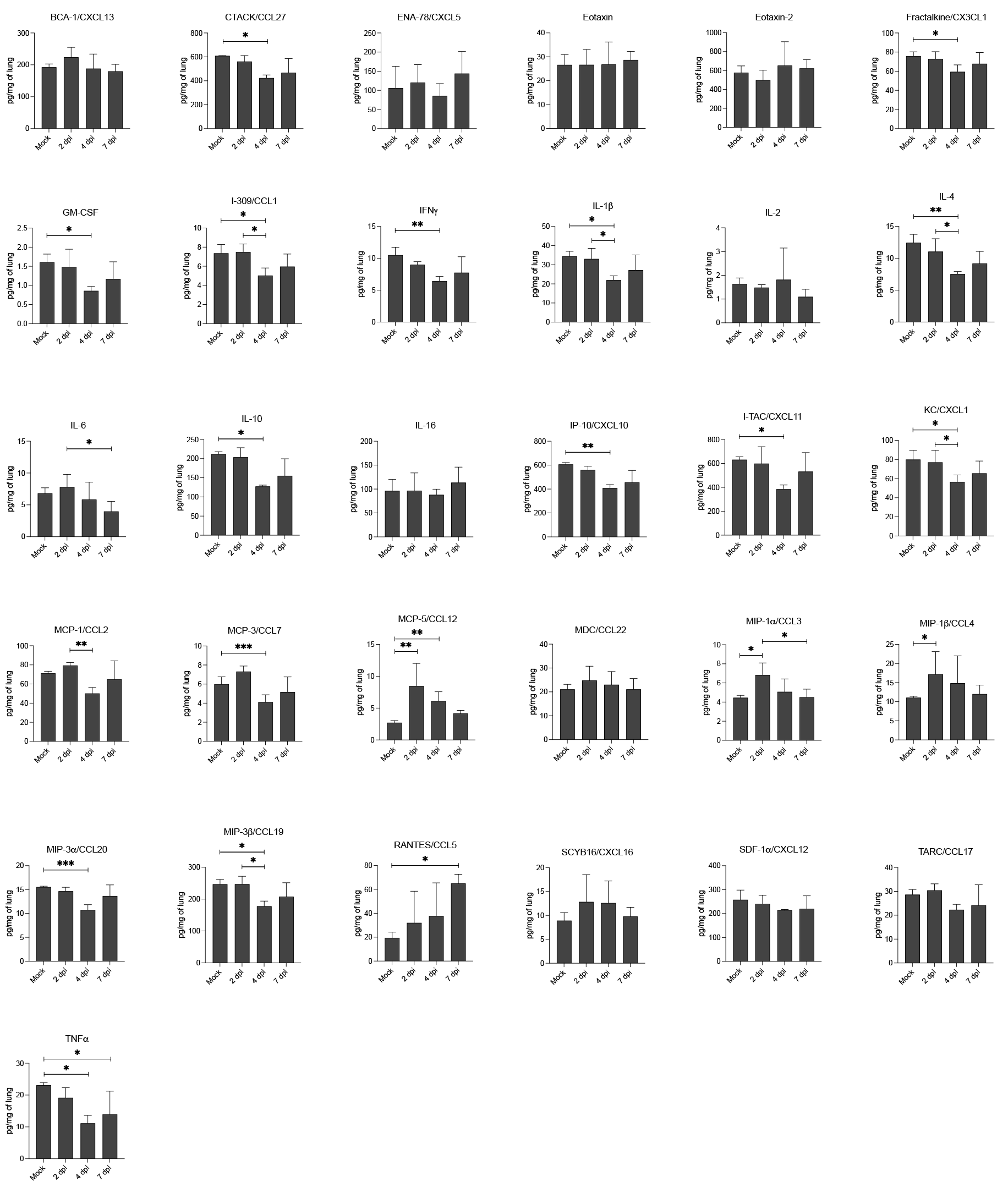

Supplement: FIG S2 [file msphere.00558-22-s0002.tif]

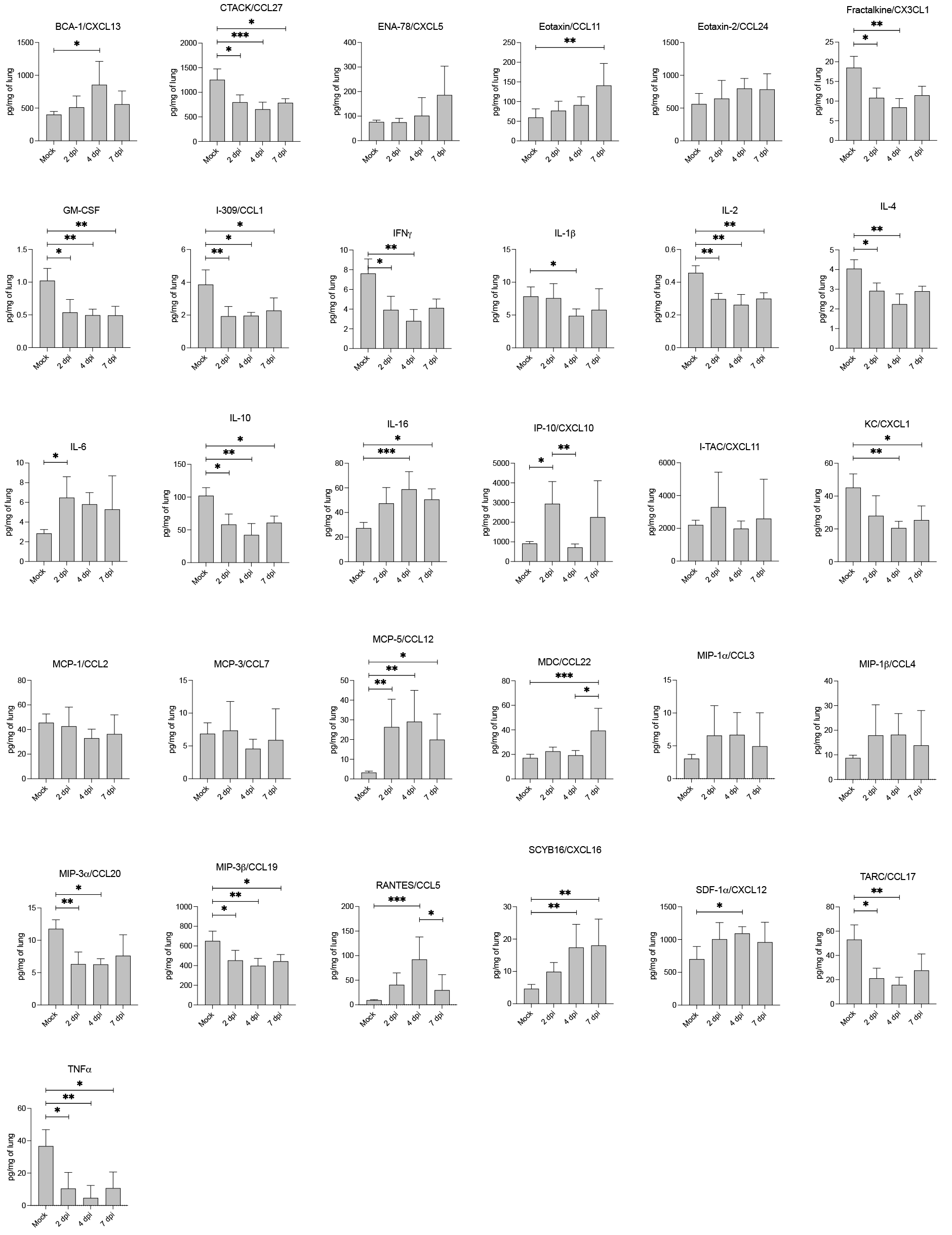

Supplement: FIG S3 [file msphere.00558-22-s0003.tif]

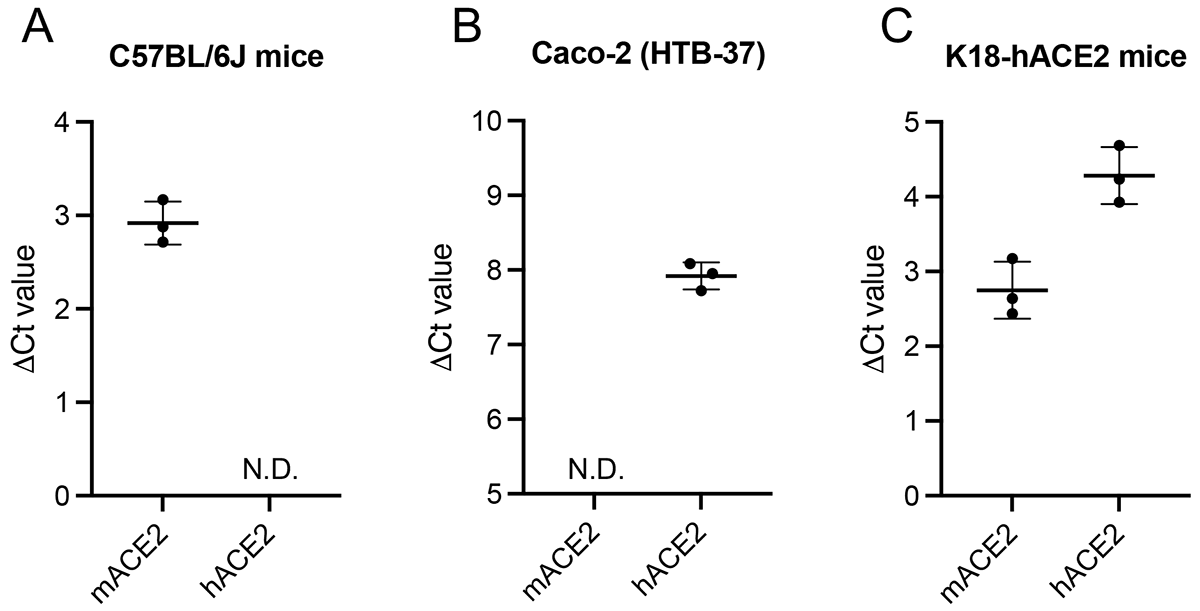

Supplement: FIG S4 [file msphere.00558-22-s0004.tif]

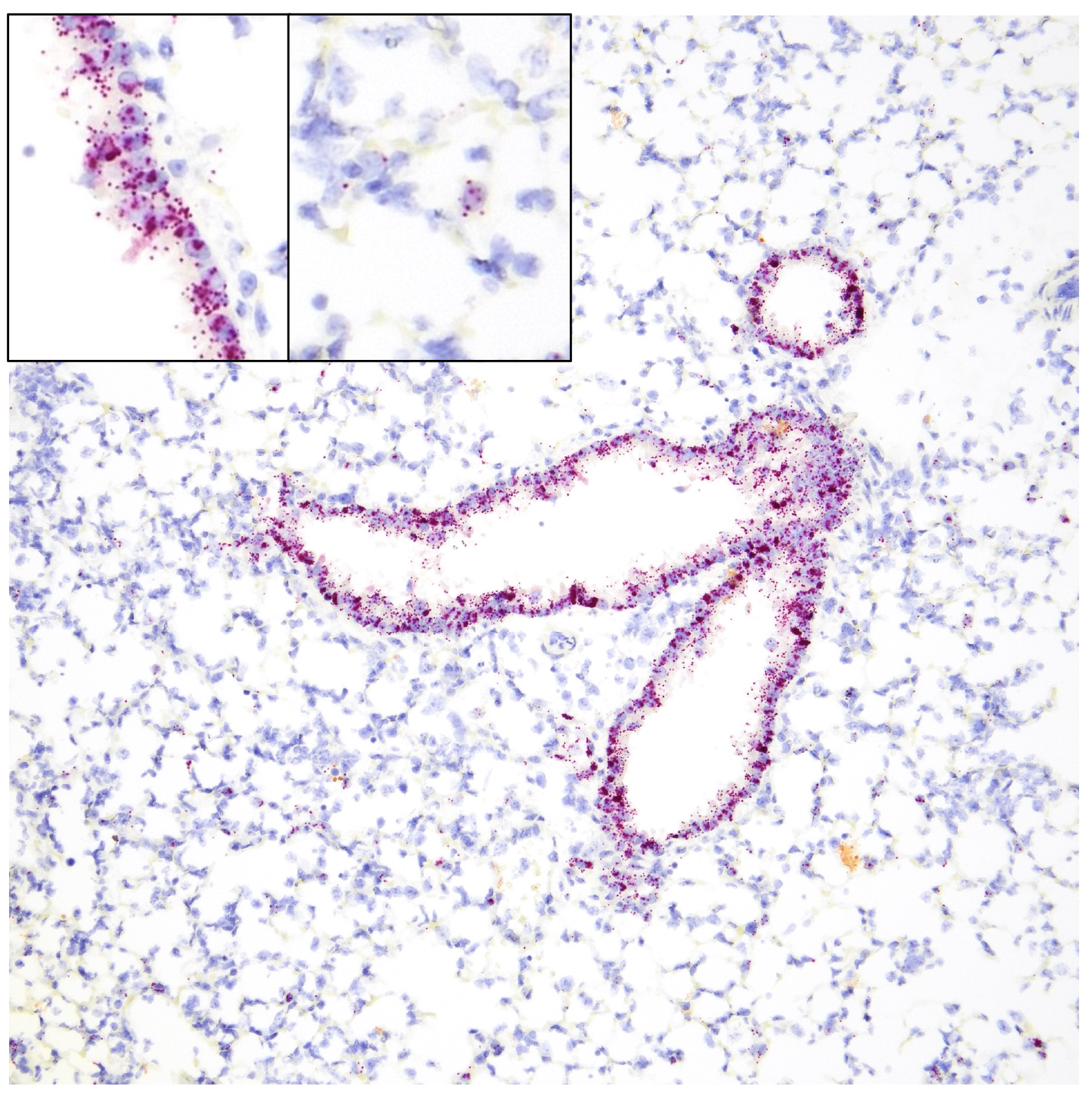

Supplement: FIG S5 [file msphere.00558-22-s0005.tif]
